# Inductive analysis of the spatial distribution characteristics of neurons that innervate skeletal muscle and their correlation with muscle phenotype

**DOI:** 10.4103/NRR.NRR-D-24-01540

**Published:** 2025-08-13

**Authors:** Xinyi Gu, Chen Huang, Shen Wang, Jin Deng, Shuhang Guo, Xiaofeng Yin

**Affiliations:** 1 Department of Trauma and Orthopedics, Peking University People’s Hospital, Beijing, China; 2 Key Laboratory of Trauma and Neural Regeneration (Peking University), Ministry of Education, Beijing, China; 3 National Center for Trauma Medicine, Beijing, China

**Keywords:** 3D imaging, dorsal root ganglia, motor neuron, retrograde tracing, skeletal muscle, sympathetic ganglion, transcriptome

## Abstract

To perform various functions in the body, skeletal muscle is controlled and coordinated as a whole by nerves. However, there has been little research into whether the nerve control characteristics of different muscles are different, and the importance of these potential differences. In the present study, we used a three-dimensional imaging of solvent-cleared organ-compatible multi-tracer technique to explore the spatial distribution patterns of sensory and sympathetic neurons that innervate limb muscles. We integrated transcriptome sequencing datasets from mouse limb muscles in public databases and performed correlation analysis with neuronal spatial distribution data to reveal the unique effects of different types of neurons on muscle functional pathways. In terms of spatial distribution patterns, sympathetic neurons exhibited a more concentrated distribution than sensory and motor neurons. In addition, the neuronal innervation of limb muscles exhibited four different characteristics: sympathetic neuron-rich muscle, sensory neuron-rich muscle, neuron-sparse muscle, and motor neuron-rich muscle. Sensory neuron density was mainly associated with muscle contractile structure and cell pH, whereas sympathetic neuron density was associated with protein kinase activity, muscle vasculature, muscle calcium-dependent protein kinase activity, lipid transport, and vesicle release. Motor neuron density was mainly associated with protein kinase activity, cell adhesion, oxidoreductase activity, and exocytosis. These findings may contribute to a deeper understanding of how nerves cooperate to endow muscles with diverse physiological functions, thereby providing new insights and experimental evidence for the treatment of various neuromuscular diseases.

## Graphical abstract

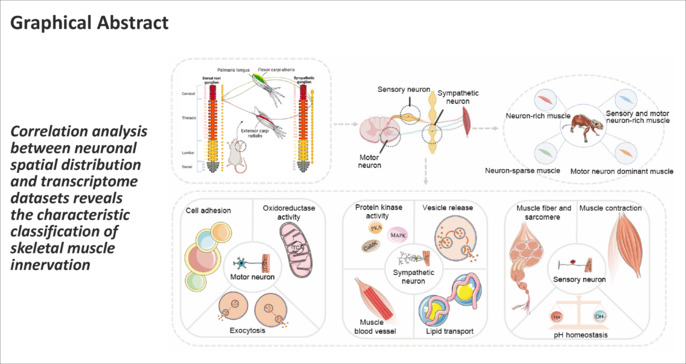

## Introduction

Skeletal muscles are a type of organ that are highly innervated and regulated by nerves. In addition to their role in movement, skeletal muscles serve as regulators of whole-body energy and substance metabolism (Etienne et al., 2020). Skeletal muscles are also crucial for overall body movement, energy supply, and fluid balance (Argiles et al., 2016; Canfora et al., 2022). Neural innervation plays an important role in maintaining muscle function. It not only enables voluntary muscle control but also helps to maintain homeostasis by regulating the balance between metabolic reactions of synthesis and breakdown (Sartori et al., 2021; D’Ercole et al., 2022). To date, research into the neural regulation of skeletal muscles has tended to focus on exploring individual muscles, and has overlooked how the skeletal muscle system as a whole is innervated and coordinated to perform different functions in the body (D’Ercole et al., 2022). Understanding how neurons participate in the various physiological functions of skeletal muscles will allow a deeper understanding of the mechanisms underlying skeletal muscle function and may provide new therapeutic ideas and a theoretical basis for the treatment of muscle diseases.

The neural network of skeletal muscles consists of sensory, motor, and sympathetic neurons. Sensory neurons innervate muscle spindles, tendons, ligaments, and joint capsules, and transmit proprioceptive impulses to the central nervous system (Meltzer et al., 2021). Spinal motor neurons control over 100 million muscle fibers in the human body to ensure coordinated muscle contractions. The α motor neurons innervate extrafusal muscle fibers and drive muscle contraction, whereas the γ and β motor neurons innervate intrafusal fibers within muscle spindles and play complex roles in motor control (Kanning et al., 2010). Traditionally, the neural regulation of skeletal muscles was believed to only involve sensory and motor neurons, and previous studies have generally reported that the sympathetic nervous system regulates skeletal muscle metabolism by controlling blood vessels (Hansen et al., 2020; DeLorey and Clifford, 2022; Rudolf et al., 2024). However, recent research indicates that the sympathetic nervous system can also directly participate in the innervation and regulation of skeletal muscle fibers (Rodrigues et al., 2019; Delbono et al., 2021), although the specific mechanisms remain unclear. Thus, the neural network of skeletal muscles, comprising sensory, motor, and sympathetic neurons, works together to participate in muscle perception, movement, and metabolic regulation. However, relatively little is known about the spatial distribution characteristics of different types of neurons in the innervation of whole-body skeletal muscles, and about the specific mechanisms by which they regulate the various physiological functions of skeletal muscles.

In recent years, although research into the neural distribution of skeletal muscles has gained increasing attention, there have only been limited systematic studies into the spatial distribution of various types of neurons that innervate muscles. The distribution of sensory and sympathetic neurons in skeletal muscles has been explored for some trunk muscles and a few limb muscles (Alpantaki et al., 2005; Chiocchetti et al., 2005; Chyczewski et al., 2006). There is therefore a need for a comprehensive and supplementary three-dimensional (3D) map of the neural network that innervates the major limb muscles throughout the body. A systematic map of this neural network is crucial as a structural foundation for exploring emerging therapies for neuromuscular diseases, whether they involve stimulating specific spinal segments or dorsal root ganglia (DRG) from the central to the peripheral nervous system (Wagner et al., 2018), the retrograde delivery of therapeutic proteins or transgenic sequences to neurons through intramuscular injections (Kemaladewi et al., 2019; Kenjo et al., 2021), or the electrical stimulation of cutaneous receptors to evoke electrical activity in muscles, the spinal cord, and the motor cortex (Merton et al., 1982).

In our previous work, we used a multiple retrograde tracing technique that is compatible with 3D imaging of solvent-cleared organ (3DISCO) to delineate a 3D spatial distribution map of motor neurons that innervate the limb skeletal muscles (Qi et al., 2022; Huang et al., 2024). In the present study, we used the same technique to explore the 3D spatial distribution of sensory and sympathetic neurons. We analyzed the characteristics of neural innervation in the major limb skeletal muscles and correlated them with skeletal muscle transcriptomic data. By examining the correlations between different types of neurons and the skeletal muscle transcriptome, we aimed to further investigate the functional regulatory mechanisms of nerves on skeletal muscles.

## Methods

### Animals

All animal care and procedures were conducted in strict accordance with the National Institutes of Health Guide for the Care and Use of Laboratory Animals (8^th^ ed., National Research Council, 2011) and were approved by the Ethics Committee of Peking University People’s Hospital Experimental Animal Management (approval no. 2020PHE089) on December 23, 2021. Twenty-seven male C57BL/6 mice, aged 6–8 weeks and weighing 20–30 g, were selected for retrograde tracing of the forelimb and hindlimb muscles. The reasons for selecting only male mice were as follows: 1) transcriptome datasets used in this study were all from male animals (C57BL/6) to ensure data consistency, and 2) literature indicates that there are no significant sex differences in basic neuroanatomy (Guma et al., 2024). The mice were purchased from Beijing Vital River Laboratory Animal Technology Co., Ltd. (license No. SYXK (Jing) 2022-0052). The relative humidity in the animal housing room was maintained at 50%–60%, and the temperature was controlled at 20–25°C. Animals were maintained on a 12-hour light/dark cycle and provided with *ad libitum* access to food and water.

### Muscle groups

Using the descriptions by Greene (1955), we determined the anatomical locations of each muscle and grouped the target muscles based on their function and position.

### Retrograde tracers

The selection of retrograde tracers was primarily based on previous work by our team (Huang et al., 2024) in which several commonly used tracers with different excitation and emission wavelengths were compared. These tracers included Fluoro-Gold (FG), Fluoro-Ruby, cholera toxin subunit B (CTB), Red-Retrobeads, Fluoro-Emerald, and Green-Retrobeads. We also determined the compatibility of these tracers with optical clearing methods and their respective labeling efficiencies (Xu et al., 2021). In the present experiment, we selected four dyes—FG, CTB-488, CTB-555, and CTB-647—for the retrograde tracing of sensory neurons and sympathetic neurons.

### Retrograde tracing process

Mice were anesthetized via inhalation of isoflurane (3% for induction, 1%–2% for maintenance in 100% O_2_; Baxter, Deerfield, IL, USA) using a precision vaporizer. Anesthesia was induced in a sealed chamber followed by maintenance via a nose cone. Body temperature was maintained at 37 ± 1°C using a heating pad, and ocular lubricant (Lacri-Lube, Allergan, Dublin, Ireland) was applied to prevent corneal drying. The limbs of the mice were prepared and sterilized following strict aseptic principles to ensure that the sterilization coverage included the surgical site. A longitudinal incision was made on the skin surface to fully expose all target muscles while preserving the integrity of the muscle fascia as much as possible. Vaseline was applied between muscle gaps to prevent tracer leakage to adjacent muscles. Using a 5-µL Hamilton microsyringe, dye was slowly injected into the muscles. The dyes comprised FG (Fluorochrome, Denver, CO, USA), CTB488 (CTB conjugated with Alexa Fluor 488, Invitrogen, Carlsbad, CA, USA), CTB555 (CTB conjugated with Alexa Fluor 555, Invitrogen), and CTB647 (CTB conjugated with Alexa Fluor 647, Invitrogen). These retrograde tracer dyes were prepared at optimal concentrations: 4% FG in 0.01 M phosphate-buffered solution, 1% CTB488 in 0.01 M phosphate-buffered solution, 1% CTB555 in 0.01 M phosphate-buffered saline, and 1% CTB647 in 0.01 M phosphate-buffered saline. All dyes were maintained on ice throughout the preparation and tracing procedures. After the muscle injection, a 5-minute waiting period was observed before the needle was removed. The muscle surface was then gently wiped with a cotton swab soaked in saline to remove any potential tracer leakage. A schematic diagram of the multiple retrograde tracing of muscles is provided in our previous study (Han et al., 2019). Finally, the wound was closed layer by layer using 5-0 sutures.

### Dorsal root ganglia and sympathetic ganglia harvest

The tracers were retained in the mice for 7 days to achieve optimal retrograde transport (Xu et al., 2021). Next, the mice were deeply anesthetized by inhalation of isoflurane (5% for induction, 1%–2% for maintenance in 100% O_2_; Baxter), followed by perfusion through the heart with physiological saline at 37°C and 4% paraformaldehyde. The collection of each segmental ganglion was based on the landmarks of the C1 nerve root (below the first cervical vertebra) and the T13 nerve root (below rib 13). Accordingly, the DRG on the C1–C8, T1–T13, C1–C6, and S1–S3 nerve roots, as well as all sympathetic ganglia (SG) located anterior and lateral to the vertebrae, were sequentially extracted. The collected DRG and SG were placed in 1.5 mL Eppendorf tubes pre-filled with 4% paraformaldehyde and were fixed overnight at 4°C in a dark environment.

### Optical clearing procedure

The fixed DRG and SG tissues were subjected to optical clearing using the 3DISCO method (Ertürk et al., 2012). The obtained tissue was sequentially immersed in a graded series of tetrahydrofuran solutions (50%, 70%, 80%, and 100% tetrahydrofuran; Aladdin, Beijing, China) for dehydration. Each immersion step lasted ≥ 20 minutes. The ganglion tissue was then transferred to Eppendorf tubes filled with dibenzyl ether (Aladdin) until the tissue became completely transparent. Prior to imaging, the ganglion tissue was stored in dibenzyl ether at 4°C in a dark environment.

### 3D imaging

The transparent ganglion tissue was subjected to 3D imaging using a laser scanning confocal microscope (SP8 DIVE, Leica, Wetzlar, Germany). The imaging parameters were as follows: bit depth = 8, pinhole = 1.0 airy units. Imaging was performed using a 10× objective. Only ganglia that exhibited fluorescent signals are displayed in the results.

### Consensus clustering

Consistency clustering analysis of the normalized neuron counts for different muscles was performed using the “ConsensusClusterPlus” package in R (https://anaconda.org/bioconda/bioconductor-consensusclusterplus). The muscle samples were divided into *k* groups using the *k*-means algorithm, with k ranging from 2–10. A cumulative distribution function plot was used to identify the value of k that had the approximate maximum distribution and indicated the highest stability. Based on the cumulative distribution function plot, *k* was determined to be 4. A clustering heatmap and a two-dimensional distribution plot were generated for the muscles using *k* = 4.

### Correlation analysis between neuron counts and muscle phenotypes

The correlation analysis between normalized neuron counts and other muscle characteristics (muscle wet weight, slow fiber composition, muscle length, fiber length, pennation angle, and physiological cross-sectional area [PCSA]) was performed using the Correlation plot app in Origin 2020b (OriginLab, Northampton, MA, USA). The data for muscle characteristics were obtained from publicly available sources (Burkholder et al., 1994; Mathewson et al., 2012; Queeno et al., 2023), whereas the muscle wet weight data was measured in the present study. Absolute Pearson’s correlation coefficient values ≥ 0.7 were considered relevant.

### Retrieval and normalization of the skeletal muscle sequencing dataset

The Gene Expression Omnibus (http://www.ncbi.nlm.nih.gov/geo/) and PubMed (http://www.ncbi.nlm.nih.gov/pubmed) were used for the dataset retrieval. The keywords used for gene expression data were as follows: “muscle,” “skeletal muscle,” and “mouse and muscle.” Our inclusion criteria were as follows: (1) gene expression data from skeletal muscles of normal C57BL/6 mice, (2) *n* ≥ 3, and (3) all gene expression analysis platforms were considered. Our exclusion criteria were as follows: (1) non-skeletal muscle samples, (2) non-mouse datasets, and (3) review studies. After consolidating the datasets, the intersection of their transcriptomes was obtained, and the non-intersecting parts of each dataset were removed. To normalize the data, the genes in each dataset were sorted based on the fragments per kilobase of transcript per million mapped reads or the count. Finally, the expression index for each gene was calculated as follows: 1 – (sorting rank / total number of genes).

### Correlation analysis between neuron counts and muscle transcriptome

The correlation analysis between normalized neuron counts and normalized gene expression indices was performed using the Correlation plot app in Origin 2020b. Absolute Pearson’s correlation coefficient values ≥ 0.7 were considered relevant.

### Gene Ontology and Kyoto Encyclopedia of Genes and Genomes enrichment analyses

Gene Ontology (GO) (http://www.geneontology.org) and Kyoto Encyclopedia of Genes and Genomes (KEGG) (http://www.genome.ad.jp/kegg/) analyses were conducted for neuron-related genes. GO terms and KEGG pathways with *P* < 0.05 were considered significantly enriched. The Database for Annotation, Visualization, and Integrated Discovery (DAVID; https://david.ncifcrf.gov/) was used for the functional annotation clustering of GO pathways; the top five clusters with the largest enrichment scores are shown. The bar charts of GO terms and KEGG pathways were drawn using Microsoft Excel 2016 (Microsoft, Redmond, WA, USA).

### Acupuncture point (acupoint) function and location labeling

The acupoint functions and location information were referenced from the *Atlas of Acupuncture* (Focks et al., 2008).

### Quantitative reverse transcription-polymerase chain reaction

Total RNA was extracted from muscle using the TRIzol method (Bo et al., 2021). DNA was removed using DNase І (Aladdin). The purity and concentration of RNA sample solutions were determined using a NanoPhotometer (IMPLEN, Munich, Germany). RNA solutions with optical density 260/280 values of 1.8–2.0 were used for the quantitative reverse transcription-polymerase chain reaction (qRT-PCR) experiments. RNA reverse transcription was performed using the 5× All-In-One RT MasterMix Kit (ABM, Vancouver, Canada). In accordance with the manufacturer’s instructions, the reagents were mixed with the DNA and incubated at 42°C for 15 minutes before the reaction was terminated at 85°C for 5 minutes to complete the reverse transcription using a Real-Time PCR System (Bio-Rad, Hercules, CA, USA). Samples were then immediately placed on ice, briefly centrifuged, and underwent PCR. The PCR reaction system included the complimentary DNA template (1–100 ng), forward/reverse primers (0.2–0.5 µM each), and SYBR Green Master Mix (TOYOBO, Osaka, Japan). The final volume was 20 µL. The reaction conditions included an initial step of 30 seconds at 95°C; 40 cycles of 5 seconds at 95°C, 30 seconds at 60°C, and 30 seconds at 72°C; and a final hold at 4°C. This process was completed using a Real-Time PCR System (Bio-Rad). The relative expression ratios of target mRNA were normalized to GAPDH expression using the 2^–ΔΔCt^ (or Livak) method (Livak and Schmittgen, 2001). Primers are listed in **Additional Table 1**.

**Additional Table 1:** Primers used for quantitative reverse transcription-polymerase chain reaction analysis

Gene	Primers (5’-3’)
Gapdh	F: AACTTTGGCATTGTGGAAGG R: ACACATTGGGGGTAGGAACA
Dmd	F: AGCTCAACCGTCGATTTGCAGC R: TTCAGCCTCCAGTGGTTCAAGC
Actn3	F: ATATCGTGAACACCCCCAAA R: TCCACTCCAACAGCTCACTG
Cav3	F: GATAGACTTGGTGAACCGCGAC R: ACTTGGAGACGGTGAACGTGGT
Stac3	F: GTCAGTCCTACGTGGAGATGCA R: GCGTACTGTTGGTCGCTGTAGA
Atp2a1	F: GAAGCCTCTCTAAAGTGGAGCG R: CGTGAGGACTTAGCTGGTGAAC
Abca1	F: GGAGCCTTTGTGGAACTCTTCC R: CGCTCTCTTCAGCCACTTTGAG
Abcg1	F: GACACCGATGTGAACCCGTTTC R: GCATGATGCTGAGGAAGGTCCT
Apoe	F: GAACCGCTTCTGGGATTACCTG
Apoe	R: GCCTTTACTTCCGTCATAGTGTC
Ldlr	F: GAATCTACTGGTCCGACCTGTC R: CTGTCCAGTAGATGTTGCGGTG
Npc1	F: TGAGGTCATCCCATTCCTGGTG R: TCCAGCGTTTCCTCCTGAAGAC

### Statistical analysis

Each experiment in the current study was performed three times independently. Results are shown as the mean ± standard error of the mean. Statistical analyses were performed using Student’s *t*-test in IBM SPSS Statistics, version 24.0 (IBM Corp., Armonk, NY, USA), and *P* < 0.05 was considered significant.

## Results

### Sensory neurons that innervate the skeletal muscles of the upper and lower limbs exhibit specific segmental distribution patterns

**Figure 1:**
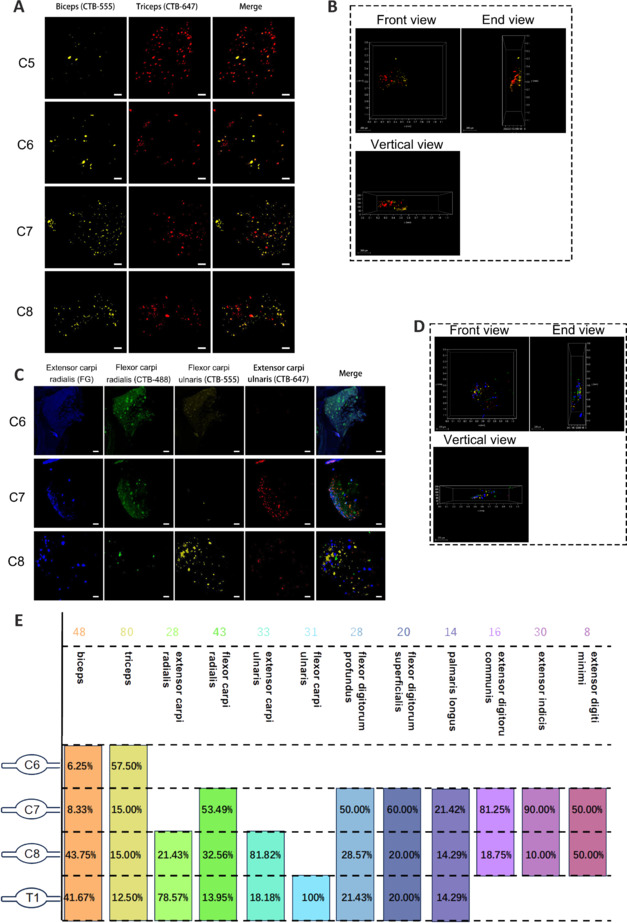
Distribution of sensory neurons in DRG that innervate upper-limb skeletal muscles. (A) DRG imaging of sensory neuron distribution segments in Group 1 (biceps and triceps). (B) Three-dimensional visualization of Group 1 representative DRG C8 from three perspectives. (C) DRG imaging of sensory neuron distribution segments in Group 2 (extensor carpi radialis, flexor carpi radialis, flexor carpi ulnaris, and extensor carpi ulnaris). (D) Three-dimensional visualization of Group 2 representative DRG C8 from three perspectives. (E) Summary diagram of the distribution of sensory neurons that innervate the upper-limb muscles, and the numbers of neurons in each segment. The number of neurons is shown at the top of each column, and the percentage is shown below. Statistical data were sourced from Figure 1 and Additional Figures 1–2. *n* = 3 per group; scale bars: 50 µm for A and C, 200 µm for B and D. 3D: Three-dimensional; CTB488 (green): cholera toxin subunit B conjugated with Alexa Fluor 488; CTB555 (yellow): cholera toxin subunit B conjugated with Alexa Fluor 555; CTB647 (red): cholera toxin subunit B conjugated with Alexa Fluor 647; FG (blue): Fluor-Gold. DRG: Dorsal root ganglia.

**Figure 2:**
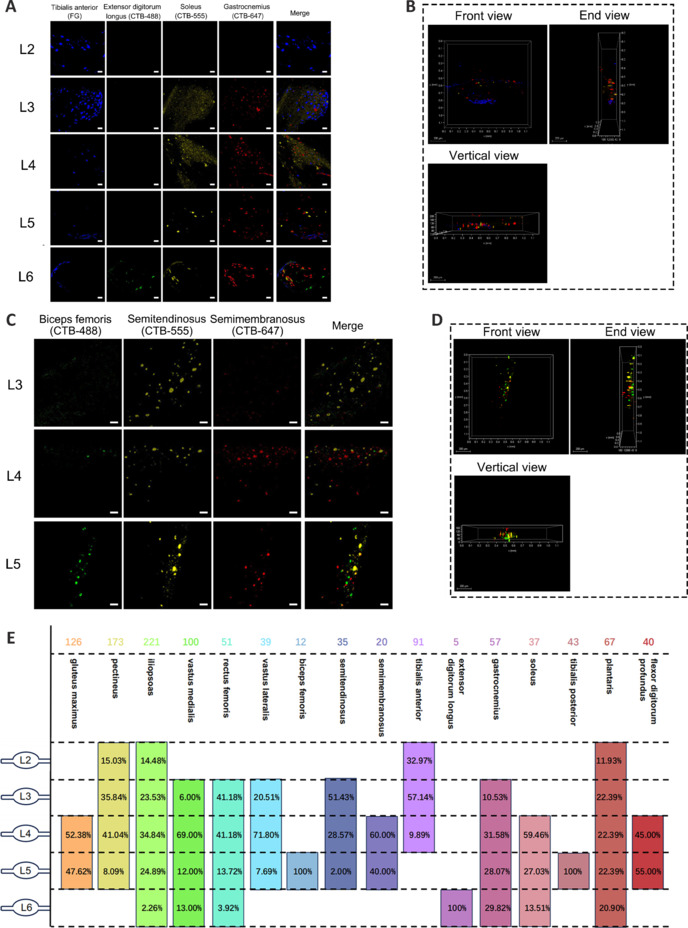
Distribution of sensory neurons in DRG that innervate lower-limb skeletal muscles. (A) DRG imaging of sensory neuron distribution segments in Group 5 (tibialis anterior, extensor digitorum longus, soleus, and gastrocnemius). (B) Three-dimensional visualization of Group 5 representative DRG L5 from three perspectives. (C) DRG imaging of sensory neuron distribution segments in Group 6 (biceps femoris, semitendinosus, and semimembranosus). (D) Three-dimensional visualization of Group 6 representative DRG L5 from three perspectives. (E) Summary diagram of the distribution of sensory neurons that innervate the upper-limb muscles, and the numbers of neurons in each segment. The number of neurons is shown at the top of each column, and the percentage is shown below. Statistical data were sourced from Figure 2 and Additional Figures 3–5. *n* = 3 per group; scale bars: 50 µm for A and C, 200 µm for B and D. 3D: Three-dimensional; CTB488 (green): cholera toxin subunit B conjugated with Alexa Fluor 488; CTB555 (yellow): cholera toxin subunit B conjugated with Alexa Fluor 555; CTB647 (red): cholera toxin subunit B conjugated with Alexa Fluor 647; FG (blue): Fluor-Gold. DRG: Dorsal root ganglia.

We first investigated the distribution of sensory neurons in DRG that innervate 12 upper-limb skeletal muscles and 16 lower-limb skeletal muscles. The muscles were categorized into nine groups based on their neural innervation or function, and multiple retrograde tracing experiments were conducted (**Additional Tables 2** and **3**). After 1 week, mouse DRG from all segments were collected and underwent tissue clearing and confocal microscopy for 3D imaging. The sensory neurons that innervated the upper-limb skeletal muscles were mainly distributed in the C6–T1 DRG (**Figure 1** and **Additional Figures 1** and **2**), whereas those that innervated the lower-limb muscles were primarily distributed in the L2–L6 DRG (**Figure 2** and **Additional Figures 3–5**).

**Additional Table 2:** Overview of multiple tracer groupings.

Nerve	Muscle
Group1	Biceps
	Triceps
	Extensor carpi radialis
Group2	Flexor carpi radialis
	Flexor carpi ulnaris
	Extensor carpi ulnaris
	Flexor digitorum profundus (upper)
Group3	Flexor digitorum superficialis
	Palmaris longus
	Extensor digitorum communis
Group4	Extensor indicis
	Extensor digiti minimi
	Tibialis anterior
	Extensor digitorum longus
	Soleus
	Gastrocnemius
Group6	Biceps femoris
	Semitendinosus
	Semimembranosus
	Rectus femoris
Group7	Vastus medialis
	Vastus lateralis
	Iliopsoas
Group8	Gluteus maximus
	Pectineus
	Plantaris
Group9	Tibialis posterior
	Flexor digitorum profundus (lower)

**Additional Table 3:** Overview of nerve *innervation in skeletal muscles*

Nerve	Muscle
	Extensor carpi radialis
	Extensor carpi ulnaris
Radial nerve	Triceps
	Biceps
	Extensor digiti minimi
	Extensor digitorum communis
	Flexor carpi ulnaris
Ulnar nerve	Flexor digitorum profundus (upper)
	Flexor carpi radialis
Median nerve	Palmaris longus
	Flexor digitorum superficialis
	Biceps femoris
	Iliopsoas
	Rectus femoris
Femoral nerve	Semimembranosus
	Semitendinosus
	Vastus lateralis
	Vastus medialis
	Soleus
	Flexor digitorum profundus (lower)
Tibial nerve	Gastrocnemius
	Plantaris
	Tibialis posterior
Inferior gluteal nerve	Gluteus maximus
	Extensor digitorum longus
Deep peroneal nerve	Tibialis anterior

### Sympathetic neurons that innervate the skeletal muscles of the upper and lower limbs exhibit specific segmental distribution patterns

**Figure 3:**
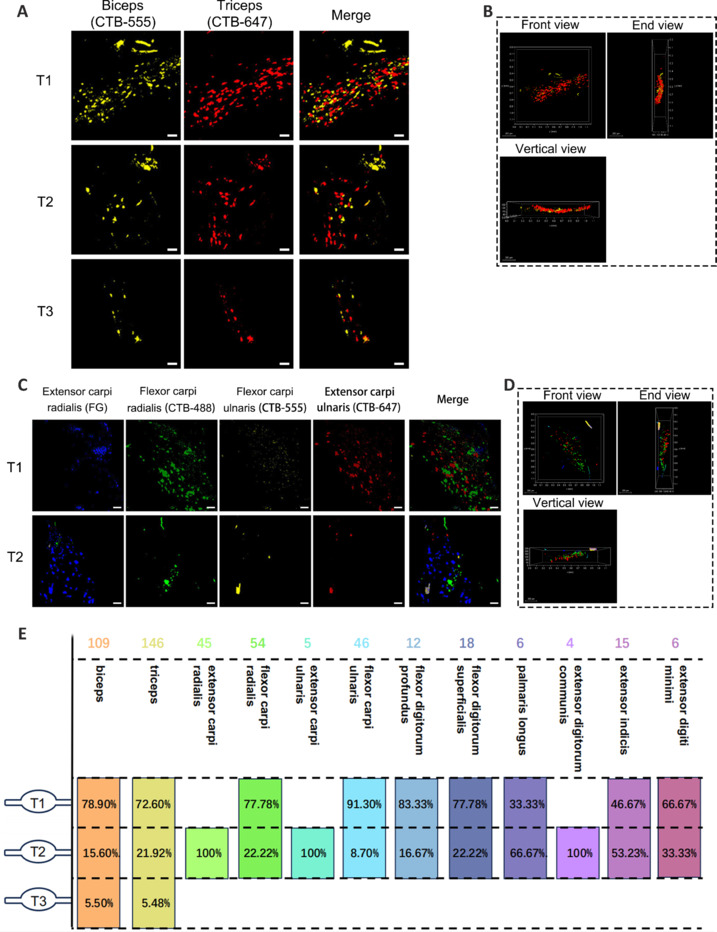
Distribution of sympathetic neurons in SG that innervate upper-limb skeletal muscles. (A) SG imaging of sympathetic neuron distribution segments in Group 1 (biceps and triceps). (B) Three-dimensional visualization of Group 1 representative SG T1 from three perspectives. (C) SG imaging of sympathetic neuron distribution segments in Group 2 (extensor carpi radialis, flexor carpi radialis, flexor carpi ulnaris, and extensor carpi ulnaris). (D) Three-dimensional visualization of Group 2 representative SG T1 from three perspectives. (E) Summary diagram of the distribution of sympathetic neurons that innervate lower-limb muscles, and the numbers of neurons in each segment. The number of neurons is shown at the top of each column, and the percentage is shown below. Statistical data were sourced from Figure 3 and Additional Figures 6–7. *n* = 3 per group; scale bars: 50 µm for A and C, 200 µm for B and D. 3D: Three-dimensional; CTB488 (green): cholera toxin subunit B conjugated with Alexa Fluor 488; CTB555 (yellow): cholera toxin subunit B conjugated with Alexa Fluor 555; CTB647 (red): cholera toxin subunit B conjugated with Alexa Fluor 647; FG (blue): Fluor-Gold. SG: Sympathetic ganglion.

**Figure 4:**
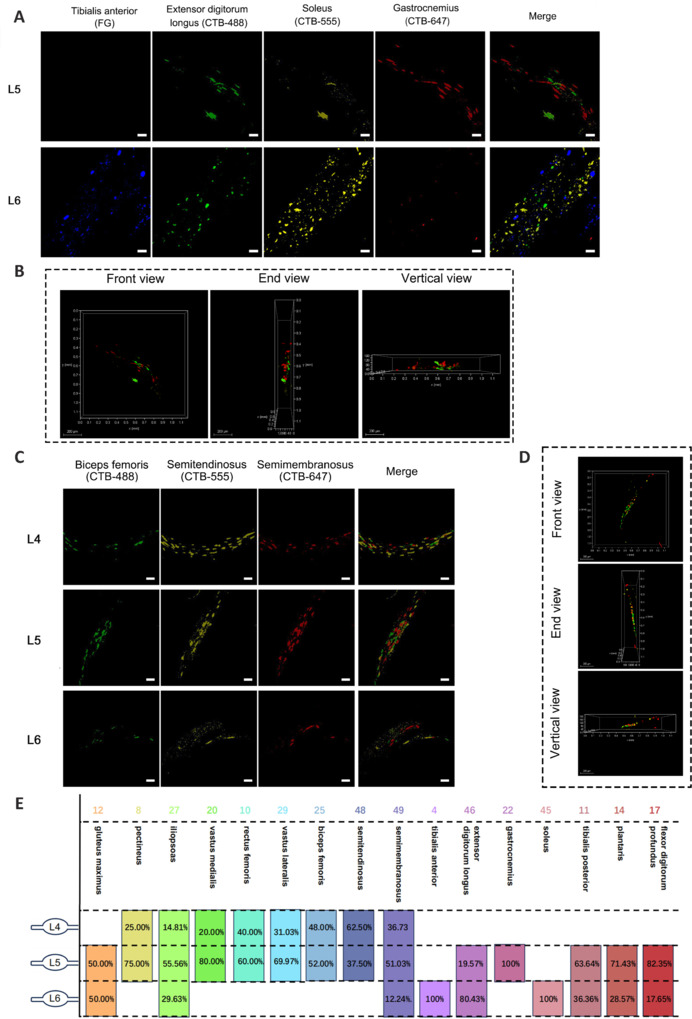
Distribution of sympathetic neurons in SG that innervate lower-limb skeletal muscles. (A) SG imaging of sympathetic neuron distribution segments in Group 5 (tibialis anterior, extensor digitorum longus, soleus, and gastrocnemius). (B) Three-dimensional visualization of Group 5 representative SG L5 from three perspectives. (C) SG imaging of sympathetic neuron distribution segments in Group 6 (biceps femoris, semitendinosus, and semimembranosus). (D) Three-dimensional visualization of Group 6 representative SG L5 from three perspectives. (E) Summary diagram of the distribution of sympathetic neurons that innervate the lower-limb muscles, and the number of neurons in each segment. The number of neurons is shown at the top of each column, and the percentage is shown below. Statistical data were sourced from Figure 4 and Additional Figures 8–10. *n* = 3 per group; scale bars: 50 µm for A and C, 200 µm for B and D. 3D: Three-dimensional; CTB488 (green): cholera toxin subunit B conjugated with Alexa Fluor 488; CTB555 (yellow): cholera toxin subunit B conjugated with Alexa Fluor 555; CTB647 (red): cholera toxin subunit B conjugated with Alexa Fluor 647; FG (blue): Fluor-Gold. SG: Sympathetic ganglion.

We next investigated the distribution of sympathetic neurons that innervate the limb muscles within SG. In mice, SG mainly consist of sympathetic chain ganglia and prevertebral and pelvic ganglia. We observed limb muscle-innervating neurons in sympathetic chain ganglia only. Sympathetic neurons that innervated the upper-limb muscles were mainly distributed in the T1–T3 SG (**Figure 3** and **Additional Figures 6** and **7**), whereas those that innervated the lower-limb muscles were primarily distributed in the L4–L6 SG (**Figure 4** and **Additional Figures 8–10**).

### Compared with sensory and motor neurons, sympathetic neurons that innervate skeletal muscles are more concentrated in segments

**Figure 5:**
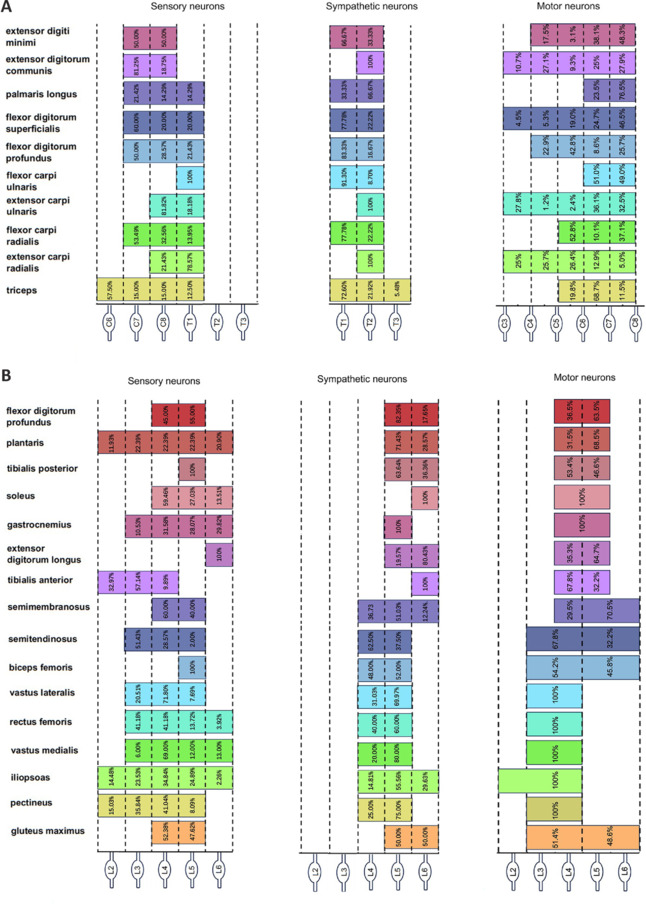
Summary diagram of the spatial distributions of the three types of neurons. (A) Neurons that innervate the upper limbs. (B) Neurons that innervate the lower limbs. *n* = 3.

We combined and analyzed the data on the distribution segments and quantities of sensory and sympathetic neurons that innervate the limb muscles, together with motor neuron data that were previously reported by our group (Qi et al., 2022; Huang et al., 2024; **Figure 5**). In terms of the spinal segment distribution of neurons, motor neurons tended to be located higher, whereas sensory neurons were in the middle and sympathetic neurons were lower. In terms of the spatial distribution pattern of neurons, sympathetic neurons were more concentrated than sensory and motor neurons, suggesting that there are differences in peripheral innervation patterns among different types of neurons. The concentrated distribution of sympathetic neurons may allow them to respond more rapidly to the needs of the body and to rapidly and precisely regulate skeletal muscles.

We also observed significant differences in the quantities of different types of neurons; however, these differences were unable to be generalized using a single rule. We therefore performed a cluster analysis to further investigate these differences.

### Skeletal muscles can be classified into four characteristic subpopulations based on their neuronal innervation patterns

**Figure 6:**
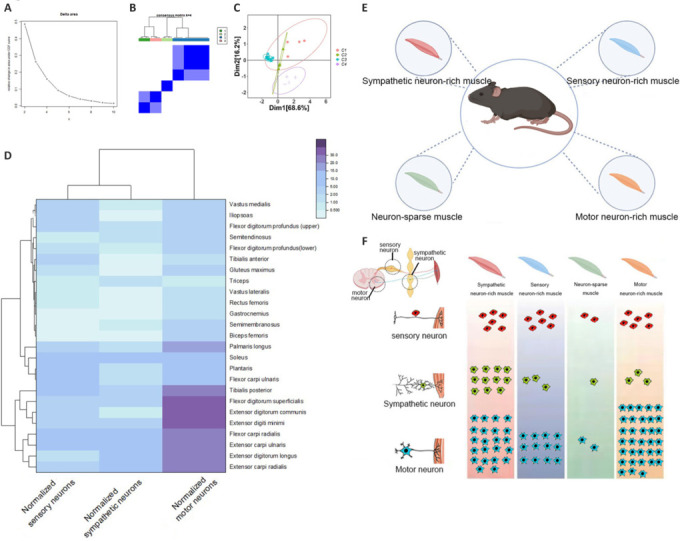
Consensus clustering of skeletal muscles based on neurons. (A) Consensus cumulative distribution function (CDF) graph (*k* = 2–10). (B) Muscle clustering diagram when *k* = 4. (C) Two-dimensional display of consistency clustering when *k* = 4. (D) Clustered heat map showing all muscles. (E) Skeletal muscles are divided into four clusters. (F) Average number of normalized neurons per cluster. *n* ≥ 3.

To eliminate bias caused by muscle mass, we normalized the quantities of sensory, sympathetic, and motor neurons by dividing them by the mass of each muscle. We then performed consensus clustering using the normalized quantities. Based on the cumulative distribution function plot, we divided the muscles into four clusters (**Figure 6A–C**). The clustering heatmap and grouping table of all muscles show the neuronal innervation characteristics (**Figure 6D–F** and **Table 1**). There were four main characteristics of muscles based on neuronal innervation; we categorized them as sympathetic neuron-rich muscle, sensory neuron-rich muscle, neuron-sparse muscle, and motor neuron-rich muscle.

**Table 1:** Classification of muscles based on three types of neurons

Cluster	Characteristic	Muscle	Normalized sensory neurons (/g)	Normalized sympathetic neurons (/g)	Normalized motor neurons (/g)
C1	Sympathetic neuron-rich muscle	Extensor carpi radialis	4.57	7.33	22.58
		Extensor carpi ulnaris	10.54	14.98	26.77
		Extensor digitorum longus	1.28	11.26	20.98
		Flexor carpi radialis	10.12	12.94	21.19
C2	Sensory neuron-rich muscle	Flexor carpi ulnaris	7.41	1.16	10.24
		Palmaris longus	3.63	1.42	17.44
		Plantaris	7.65	1.58	10.23
		Soleus	5.51	6.67	10.88
C3	Neuron-sparse muscle	Biceps femoris	0.10	0.23	2.34
		Flexor digitorum profundus (lower)	1.43	0.61	4.70
		Flexor digitorum profundus (upper)	3.68	1.61	4.61
		Gastrocnemius	0.47	0.18	1.03
		Gluteus maximus	1.44	0.14	2.64
		Iliopsoas	4.01	0.19	2.86
		Rectus femoris	0.83	0.16	1.21
		Semimembranosus	0.23	0.59	2.08
		Semitendinosus	0.75	1.03	3.16
		Tibialis anterior	2.03	0.09	1.67
		Triceps	0.76	1.38	0.91
		Vastus lateralis	0.61	0.46	1.32
		Vastus medialis	3.56	0.72	2.80
C4	Motor neuron-rich muscle	Extensor digiti minimi	4.12	2.89	31.5
		Extensor digitorum communis	3.85	0.84	33.33
		Flexor digitorum superficialis	3.15	2.94	39.19
		Tibialis posterior	13.08	3.20	27.88

**Figure 7:**
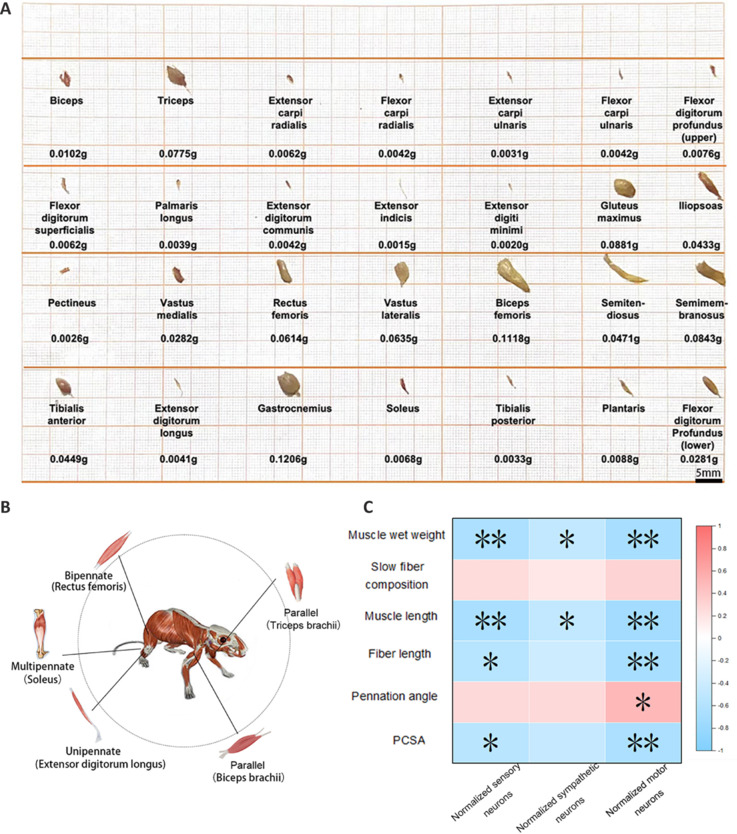
Correlation analysis between neuron number and other muscle representations. (A) Morphology and muscle weight of major skeletal muscles in mice. (B) Muscles have different fiber characteristics. (C) Correlation analysis results. The colors represent Pearson’s correlation coefficients; blue are negative correlations and red are positive correlations. *n* ≥ 3 per group; scale bar: 5 mm for A. Data are expressed as the mean ± standard error of the mean; *P < 0.05, **P < 0.01.

To further analyze the physiological importance of neuronal innervation characteristics, we measured muscle weight and retrieved data from published studies on the slow fiber composition, muscle length, fiber length, pennation angle, and PCSA of skeletal muscle (Burkholder et al., 1994; Mathewson et al., 2012; Queeno et al., 2023; **Table 2**). We then conducted correlation analyses between the normalized neuron quantities and the aforementioned muscle properties (**Figure 7**). We observed weak positive correlations between the three neuron types and the percentage of slow muscle fibers; however, this relationship did not reach significance. There was a strong correlation between the mechanical properties of muscles and neuronal density. Specifically, with increasing neuronal density innervating a muscle, the pennation angle of the skeletal muscle also increased, whereas muscle weight, muscle length, fiber length, and physiological cross-sectional area showed a decreasing trend. It is also worth noting that muscles in clusters C1, C2, and C4, with higher neuronal density, generally had larger pennation angles than those of neuron-sparse muscles, indicating a higher capacity for force generation. This finding suggests that muscles with higher neuronal density per unit mass may have greater load-bearing capacity.

**Table 2:** Summary of muscle fiber composition and mechanical data

Muscle	Muscle wet weighth (g)	Slow fiber composition (%)	Muscle length (mm)	Fiber length (mm)	Pennation angle (degree)	PCSA (mm^2^)
Extensor carpi radialis	0.0062	26.0	7.76	5.60	26.40	1.26
Extensor carpi ulnaris	0.0031	38.8	6.49	2.74	20.80	1.72
Extensor digitorum longus	0.0041	10.9	12.20	6.20	8.30	1.80
Flexor carpi radialis	0.0042	29.2	7.52	3.67	15.02	1.27
Flexor carpi ulnaris	0.0042	38.6	8.19	3.80	20.95	2.29
Palmaris longus	0.0039	43.9	5.96	3.62	24.65	0.91
Plantaris	0.0088	17.7	13.50	5.30	14.30	2.80
Soleus	0.0068	69.4	11.20	8.10	8.50	0.90
Biceps femoris	0.1118	21.0	19.00	15.00	0	11.30
Flexor digitorum profundus (lower)	0.0281	19.3				
Flexor digitorum profundus (upper)	0.0076	21.5				
Gastrocnemius	0.1206	25.4				
Gluteus maximus	0.0881	34.1				
Iliopsoas	0.0433	46.7				
Rectus femoris	0.0614	17.7	14.20	6.10	13.20	11.4
Semimembranosus	0.0843	21.2	18.60	17.10	0	1.20
Semitendinosus	0.0471	20.4				
Tibialis anterior	0.0449	16.9	12.90	7.90	11.70	5.30
Triceps	0.0775	24.5				
Vastus lateralis	0.0635	16.4	15.40	9.10	7.20	8.50
Vastus medialis	0.0282	19.7	15.30	9.00	7.20	8.60
Extensor digiti minimi	0.0020	59.6				
Extensor digitorum communis	0.0042	24.0	7.81	3.84	18.56	0.67
Flexor digitorum superficialis	0.0062	40.2	9.99	2.50	18.96	2.84
Tibialis posterior	0.0033	18.5	8.00	5.10	3.20	0.40

### Different types of neurons are associated with distinct muscle functions

**Figure 8:**
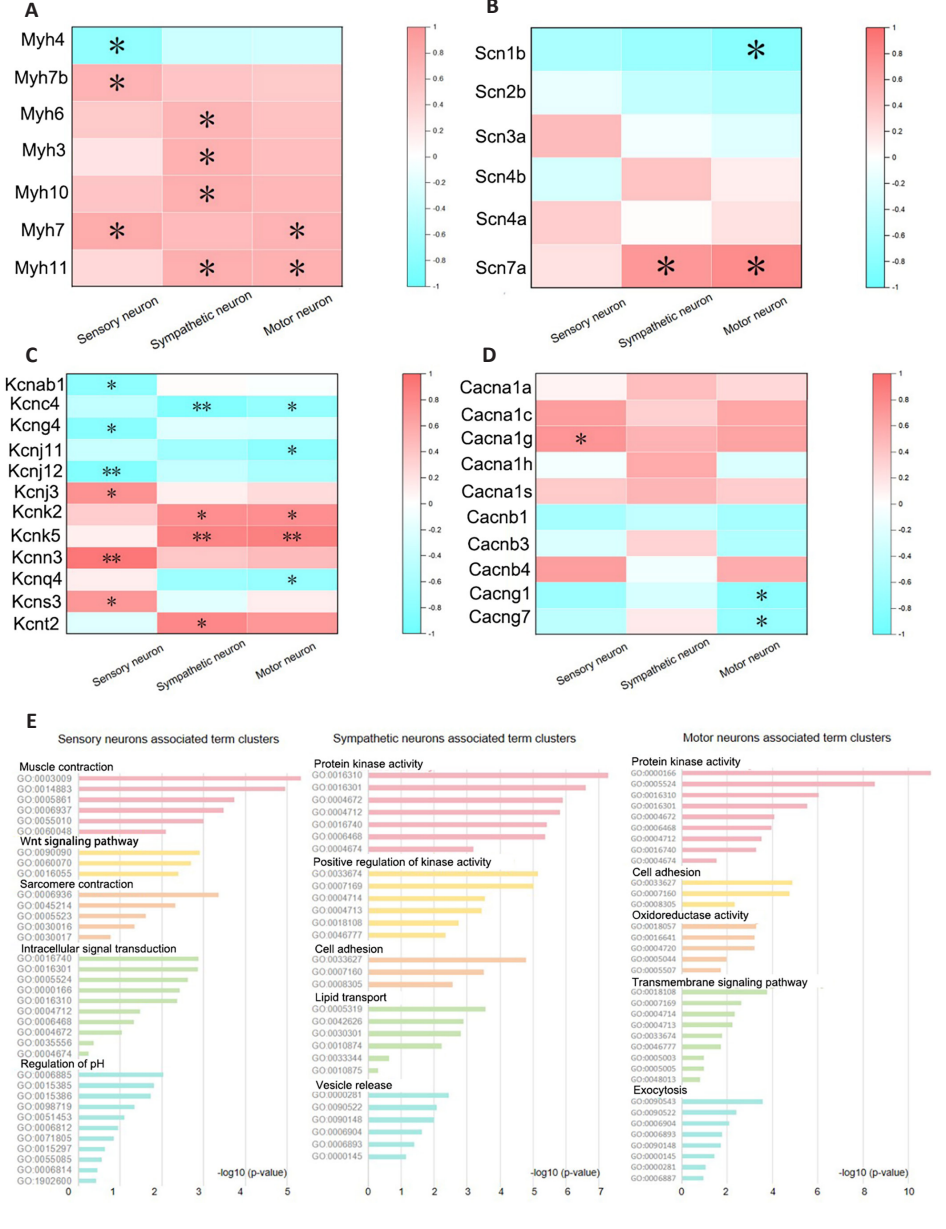
Correlation analysis between neuron number and muscle transcriptome. (A–D) Correlation analysis results of neurons with genes encoding myofibers and genes encoding ion channels. The colors represent Pearson’s correlation coefficients; blue are negative correlations and red are positive correlations. (E) The top five clusters of enriched Gene Ontology term clusters are shown for each neural positively associated gene. Data are expressed as the mean ± standard error of the mean; *P < 0.05, **P < 0.01.

We retrieved data from public databases and compiled transcriptomic datasets from normal mouse limb muscles (**Table 3**). We normalized these data and conducted a correlation analysis with neuronal density (**Additional Table 4**). **Figure 8A–D** shows the correlations between genes that encode different muscle fiber types, major ion channels (sodium, potassium, and calcium), and neurons. The densities of the three types of neurons were negatively correlated with the expression of *Myh7*, which encodes slow muscle fibers, and there was also a negative correlation between sensory neurons and *Myh4*, which encodes fast muscle fibers. These findings are consistent with the correlation trend between neuronal density and the slow muscle fiber proportion. In terms of ion channels, potassium channels were widely regulated by neurons, whereas sodium and calcium ion channels were less affected.

**Table 3:** Description of muscle transcriptome public data sets retrieved in the study

Gene expression platforms	Species	Muscle	Reference
Illumina NovaSeq 6000	Mus musculus	Gastrocnemius	GSE217576
Illumina NovaSeq 6000	Mus musculus	Tibialis anterior	GSE212352
BGISEQ-500	Mus musculus	Gluteus maximus	GSE197356
Illumina NextSeq 500	Mus musculus	Triceps	GSE233340
Illumina NextSeq 500	Mus musculus	Extensor digitorum longus	GSE158283
Illumina NextSeq 500	Mus musculus	Soleus	GSE158283
Illumina NovaSeq 6000	Mus musculus	Soleus	GSE212352
Affymetrix Mouse Gene 1.0	Mus musculus	Plantaris	GSE127255

We then performed GO enrichment analysis on genes that were positively correlated with each type of neuron. **Figure 8E** displays the top five enriched pathway clusters for each type of neuron. Sensory neurons were mainly associated with the basic structure of muscles (muscle fibers and sarcomeres), contraction activities, and pH homeostasis. Sympathetic neurons were mainly associated with protein kinase activity, muscle vasculature, muscle calcium-dependent protein kinase activity, lipid transport, and vesicle release. Motor neurons were mainly associated with protein kinase activity, cell adhesion, oxidoreductase activity, and exocytosis.

**Figure 9:**
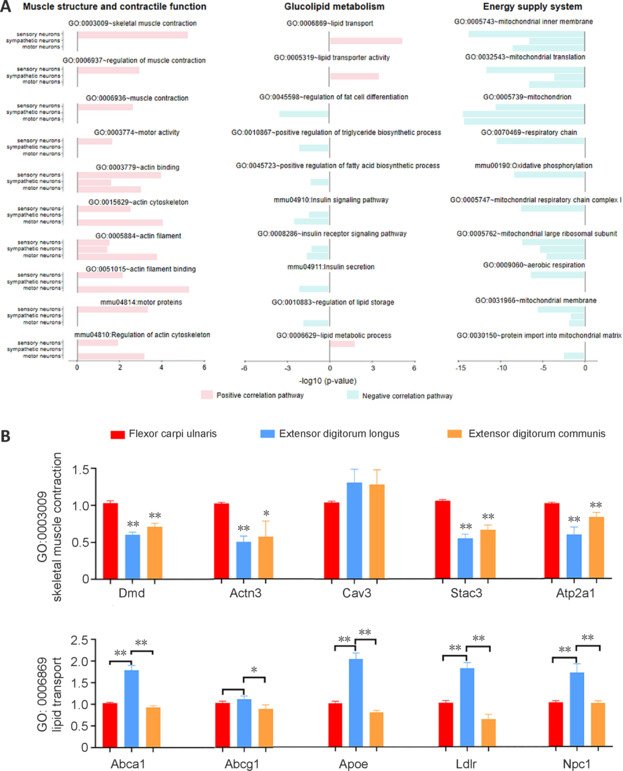
Correlation analysis between neurons and pathways for major muscle functions. (A) The left column shows muscle structure and contraction function, the middle column shows glucolipid metabolism, and the right column shows the energy supply system. (B) Quantitative reverse transcription-polymerase chain reaction detection of the relative expression levels of key genes in GO:0003009 and GO:0006869 in different muscles. Data are expressed as mean ± SEM; *P < 0.05, **P < 0.01.

Next, we investigated the correlation between the three types of neurons and the major functions of muscles, including muscle structure and contractile function, glucolipid metabolism, and energy supply system (**Figure 9A**). All three types of neurons were positively associated with muscle structure and contraction, including muscle contraction, actin and myosin activity, and motor protein activity, with sensory neurons playing a prominent role. Additionally, the glycolipid metabolism function of skeletal muscle was mainly related to sympathetic neurons. Sympathetic neurons showed a strong positive correlation with skeletal muscle lipid transport, the negative regulation of adipocyte differentiation, triglyceride and fatty acid synthesis, and insulin signaling pathways. Furthermore, energy supply in skeletal muscles was negatively correlated with all three types of neurons.

To further confirm these results, qRT-PCR was used to detect the relative expression of key genes in GO:0003009 and GO:0006869 in different muscles. We selected the flexor carpi ulnaris (a sensory neuron-rich muscle), extensor digitorum longus (a sympathetic neuron-rich muscle), and extensor digitorum communis (a motor neuron-rich muscle), which all have similar masses and can therefore be relatively easily compared. Most of the key genes in the GO:0003009 pathway (*Dmd*, *Actn3*, *Cav3*, *Stac3*, and *Atp2a1*) had high expression in the flexor carpi ulnaris and relatively low expression in the other muscles. Furthermore, most of the key genes in the GO:0006869 pathway (*Abca1*, *Abcg1*, *Apoe*, *Ldlr*, and *Npc1*) had high expression in the extensor digitorum longus and relatively low expression in the other muscles (**Figure 9B**).

## Discussion

To date, there are a lack of direct correlations between neuronal innervation and gene expression in muscles, which limits our understanding of how muscles respond to physiological and pathological contexts based on different neural innervations. By combining spatial distribution data from neurons with transcriptomic sequencing data and conducting correlation analyses, we can gain a deeper understanding of how skeletal muscles are regulated by nerves to perform various physiological functions and maintain the overall coordination of major physiological processes in the body.

Findings from the present study suggest that sensory neurons that innervate upper-limb muscles are mainly distributed in the C6–T1 DRG, whereas sensory neurons that innervate lower-limb muscles are mainly distributed in the L2–L6 DRG. This distribution pattern closely corresponds to the anatomy and function of the spinal nerves (Münzberg et al., 2023; Sonoo, 2023), and may imply a close functional relationship between motor control and sensory information processing. Through their incoming fibers, sensory neurons in the DRG are capable of sensitively perceiving muscle stretch and tension. They can then precisely adjust motor output based on the biomechanical characteristics of the muscles. These fibers also establish intricate and specific connections with neural circuits in the central nervous system (Balaskas et al., 2020; Meltzer et al., 2021; Adidharma et al., 2022), ensuring that sensory feedback can be rapidly and accurately transmitted to the motor circuits that control relevant muscle activities, thereby providing a foundation for precise motor control (Mears and Frank, 1997; Mendelsohn et al., 2015). Furthermore, single-cell transcriptomic analysis and the retrograde labeling of specific muscle groups have revealed that proprioceptive sensory neurons in the DRG are endowed with key molecular features for controlling their respective muscle types during early developmental stages (Dietrich et al., 2022). These results further emphasize the close relationship between the distribution pattern of sensory neurons and the processes of muscle development and differentiation.

Our findings indicate that sympathetic neurons that innervate upper-limb muscles are distributed in the T1–T3 SG, whereas sympathetic neurons that innervate lower-limb muscles are primarily distributed in the L4–L6 SG. However, SG segments corresponding to upper-limb muscles did not directly correspond to the respective muscle segments, which is possible because of the unique neural anatomy of the upper sympathetic trunk. In the neck region, there are only superior, middle, and inferior cervical ganglia, and the inferior cervical ganglion and T1 SG often merge to form the stellate ganglion (Piraccini et al., 2025). The stellate ganglion extensively innervates multiple organs, including the head, neck, upper limbs, and heart. A stellate ganglion block is commonly used in clinical practice to alleviate complex regional pain syndromes in the head and upper limbs (Narouze, 2014; Gunduz and Kenis-Coskun, 2017; Wie et al., 2021). Our results indicate that the majority of sympathetic neurons that innervate upper-limb muscles are located in the T1 SG, which further confirms the critical role of the stellate ganglion. The distribution of lower-limb sympathetic neurons was more concentrated than that of sensory and motor neurons, which may allow sympathetic neurons to rapidly respond to the needs of the body and quickly and precisely regulate skeletal muscles (Rodrigues et al., 2019; Delbono et al., 2021).

On the basis of the consistent clustering results of neuronal innervation in muscles, we identified four main neuronal innervation characteristics in mouse limb muscles: sympathetic neuron-rich muscle, sensory neuron-rich muscle, neuron-sparse muscle, and motor neuron-rich muscle. By contrast, recent classification studies of skeletal muscles have primarily focused on muscle fiber types and their mechanical properties (Zierath and Hawley, 2004; Schiaffino, 2010; Schiaffino and Reggiani, 2011). Different skeletal muscles exhibit diverse properties and functions because of differences in muscle weight, length, fiber length, and pennation angle (Burkholder et al., 1994; Eng et al., 2008; Mathewson et al., 2012; Ramalingasetty et al., 2021). Our experimental results indicated that muscles in the C1, C2, and C4 clusters, which had high neuronal density, mostly had larger pennation angles than those of neuron-sparse muscles (**Table 4**). This finding suggests that these muscles possess greater force-generating capacities, because a higher neuronal density per unit mass may indicate a greater load-carrying capacity (Strasser et al., 2013). The pennation angle refers to the angle between muscle fibers and the tendon (or skeletal structure) into which they insert. Muscles with larger pennation angles generally have higher force-generating capacities because more muscle fibers can simultaneously contribute to contraction, thus increasing the overall muscle force. These muscles with high neuronal density mostly have a smaller mass. Smaller muscles imply faster contraction speed and better endurance and recovery capabilities, making them more adaptable to high-frequency movements (Ross and Wakeling, 2021). The higher number of neurons per unit mass in these muscles suggests that more neurons are involved in the contraction and relaxation processes of the muscles, which likely enhances the synergy among muscle fibers. This synergy may then lead to a tighter arrangement of muscle fibers, resulting in an increased pennation angle, allowing the muscles to generate force more efficiently. Furthermore, we noted that with increased neuronal density, there was decreased muscle weight and length, fiber length, and PCSA, possibly because of a denser arrangement of muscle fibers and improved contraction efficiency. Together, these findings suggest that, while maintaining the same or higher force, muscles may improve energy efficiency by reducing their mass and volume. However, these explanations and speculations require further experimental research.

**Table 4:** Mean values of muscle mechanics data for each cluster

Cluster	Characteristic	Average wet weighth (g)	Average pennation angle (degree)
C1	Sympathetic neuron-rich muscle	0.0044	17.63
C2	Sensory neuron-rich muscle	0.0059	17.10
C3	Neuron-sparse muscle	0.0620	7.86
C4	Motor neuron-rich muscle	0.0039	13.57

To investigate the regulatory mechanisms of different types of neurons on muscle, we retrieved public transcriptomic datasets from mouse skeletal muscle (Hettige et al., 2020; Stantzou et al., 2021; Cao et al., 2022; Bittel et al., 2024; Jaime et al., 2024). We conducted correlation analyses between these data and neuron density data to identify genes associated with different neuron types. The neurons exhibited a similar relationship trend with muscle fiber types and genes encoding ion channels. Neuron density was negatively correlated with the expression of genes encoding slow-twitch muscle fibers, consistent with the correlation trend between neuron density and the proportion of slow-twitch muscle fibers. Moreover, this finding aligns with previous research indicating an increase in the proportion of slow-twitch muscle fibers as a result of denervation (Kostrominova et al., 2005).

Interestingly, the three types of neurons exhibited different characteristics in other aspects. Our GO analysis of the correlated genes indicated that genes associated with sensory, sympathetic, and motor neurons play pivotal roles in regulating muscle structure, function, and metabolism. Our findings also suggest that sensory neurons are crucial for controlling muscle fibers, motor units, and contraction activity, as well as for maintaining pH homeostasis through mechanisms involving H^+^ ion balance and respiratory reflexes (Proske and Gandevia, 2012; Guyenet and Bayliss, 2015; Mendelsohn et al., 2015; Tanaka et al., 2016; Balaskas et al., 2020; Meltzer et al., 2021; Dietrich et al., 2022). Notably, the connection between sensory neurons and pH regulation remains underexplored, offering a promising research direction. Our analysis also indicates that sympathetic neurons influence protein kinase activity, muscle vasculature, and lipid transport, and regulate muscle strength, blood flow, and metabolism via pathways including cyclic AMP/protein kinase A and calcium-dependent protein kinase (Martin, 1996; Ranallo and Rhodes, 1998; Ma et al., 2011). They also modulate vesicle release at neuromuscular junctions, which is crucial for maintaining neuromuscular junction morphology and function (Wang et al., 2020; Delbono et al., 2021; Straka et al., 2021). Motor neurons regulate protein kinase activity, cell adhesion, and oxidoreductase function, controlling muscle contractions through neuromuscular junctions and energy metabolism via oxidative phosphorylation (Wu et al., 2010; Tintignac et al., 2015; Li et al., 2018; Graham, 2022). Our bioinformatic analyses therefore support the link between motor neuron diversity and muscle fiber properties, emphasizing the intricate interplay between neurons and muscle function. The peripheral sympathetic nervous system of mice can reportedly ameliorate age-related sarcopenia by regulating the sympathetic and motor nerve innervation of skeletal muscles, thus improving acetylcholine receptor stability and muscle strength production and inhibiting the increase of inflammatory markers (Rodrigues et al., 2021a, b).

We next examined the link between the three neuron types and muscle functions, including structure, contraction, glucolipid metabolism, and energy supply. All three neuron types were positively correlated with muscle structure and contraction, with sensory neurons playing a leading role. This included muscle actin and motor protein activity, suggesting that sensory neurons guide skeletal muscle assembly and regulate growth. Furthermore, skeletal muscle glucolipid metabolism, involving lipid transport, adipocyte regulation, and insulin signaling (Nonogaki, 2000), was mainly tied to sympathetic neurons. Sympathetic neurons likely promote fat mobilization and inhibit fat synthesis, thereby finely regulating whole-body metabolism. In addition, muscle energy supply, primarily involving mitochondrial function, was negatively correlated with all three neuron types (particularly sensory neurons). Mitochondria are vital for ATP production, and although they are influenced by hormones and have been linked to exercise and aging, the neural regulation of mitochondria requires further study (Lanza and Sreekumaran Nair, 2010; Porter and Wall, 2012). We must note that the transcriptomic data in the present study came from mice aged 6–8 weeks, which means that the identified pattern of neural innervation and its relationship with muscle transcriptome may be unique to young adult mice and might change with age.

The present study has some limitations. First, although we normalized the sequencing data, potential biases from different sequencing platforms or depths may influence the results, and any potentially causal relationships between neuronal innervation and muscle gene expression need to be experimentally validated. Future studies should use single-cell sequencing combined with neural tracing techniques to confirm our findings across different muscle groups and explore the molecular mechanisms of neuromuscular interactions. Second, all current data were obtained from young adult mice, meaning that the plasticity of neuronal distribution patterns during aging or disease states remains unknown. Subsequent research should incorporate age-stratified cohorts to investigate the relationship between neuronal reorganization and muscle degeneration. Third, this study did not detect whether the retrograde tracer had diffused into non target muscle. Finally, although the co-localization of acupoints with high neuronal density muscles may have potential diagnostic and therapeutic value, direct evidence linking these anatomical features to neurophysiological effects or clinical outcomes remains lacking. Intervention studies (e.g., selective nerve blockade or activation) are needed to validate their biological basis. Such investigations will facilitate the translation of neuronal innervation characteristics into applications for muscle physiology regulation and precision medicine.

In conclusion, the present study revealed four main findings. First, in terms of spatial distribution patterns, motor neurons tended to be located higher, sensory neurons were located in the middle, and sympathetic neurons were located lower. Furthermore, compared with sensory and motor neurons, sympathetic neurons exhibited a more concentrated distribution. Second, the neuronal innervation of limb muscles exhibited four characteristics: sympathetic neuron-rich muscle, sensory neuron-rich muscle, neuron-sparse muscle, and motor neuron-rich muscle. Additionally, the neuronal density of muscles was correlated with muscle fiber type and mechanical properties, and muscles with higher neuronal density per unit mass may exhibit greater load-bearing capacities. Third, sensory neuron density was mainly associated with muscle contractile structure and cell pH; sympathetic neuron density was mainly associated with protein kinase activity, muscle vasculature, muscle calcium-dependent protein kinase activity, lipid transport, and vesicle release; and motor neuron density was mainly associated with protein kinase activity, cell adhesion, oxidoreductase activity, and exocytosis. Fourth, acupoints that co-localized with muscles rich in neuronal innervation also exhibited related neural regulatory functions. This finding may have applications in terms of the extraction of neuronal innervation characteristics for diagnosis and treatment; however, the potential existence of a mechanistic correlation requires further exploration.

## Additional files:

***Additional Table 1:*** *Primers used for quantitative reverse transcription-polymerase chain reaction analysis.*

***Additional Table 2:*** *Overview of multiple tracer groupings.*

***Additional Table 3:*** *Overview of nerve innervation in skeletal muscles.*

***Additional Table 4:*** *List of neuron-related genes.*

Additional Table 4 List of neuron-related genes

***Additional Figure 1:*** *Dorsal root ganglia (DRG) imaging of sensory neuron distribution segments in Group 3: Flexor digitorum profundus (upper), flexor digitorum superficialis, and palmaris longus.*

Additional Figure 1 Dorsal root ganglia (DRG) imaging of sensory neuron distribution segments in Group 3: Flexor digitorum profundus (upper), flexor digitorum superficialis, and palmaris longus.

***Additional Figure 2:*** *Dorsal root ganglia (DRG) imaging of sensory neuron distribution segments in Group 4: Extensor digitorum communis, extensor indicis, and extensor digiti minimi.*

Additional Figure 2 Dorsal root ganglia (DRG) imaging of sensory neuron distribution segments in Group 4: Extensor digitorum communis, extensor indicis, and extensor digiti minimi.

***Additional Figure 3:*** *Dorsal root ganglia (DRG) imaging of sensory neuron distribution segments in Group 7: Rectus femoris, vastus medialis, and vastus lateralis.*

Additional Figure 3 Dorsal root ganglia (DRG) imaging of sensory neuron distribution segments in Group 4: Extensor digitorum communis, extensor indicis, and extensor digiti minimi.

***Additional Figure 4:*** *Dorsal root ganglia (DRG) imaging of sensory neuron distribution segments in Group 8: Iliopsoas, gluteus maximus, and pectineus.*

Additional Figure 4 Dorsal root ganglia (DRG) imaging of sensory neuron distribution segments in Group 4: Extensor digitorum communis, extensor indicis, and extensor digiti minimi.

***Additional Figure 5:*** *Dorsal root ganglia (DRG) imaging of sensory neuron distribution segments in Group 9: Plantaris, tibialis posterior, and flexor digitorum profundus (lower).*

Additional Figure 5 Dorsal root ganglia (DRG) imaging of sensory neuron distribution segments in Group 4: Extensor digitorum communis, extensor indicis, and extensor digiti minimi.

***Additional Figure 6:*** *Sympathetic ganglia (SG) imaging of sympathetic neuron distribution segments in Group 3: Flexor digitorum profundus (upper), flexor digitorum superficialis, and palmaris longus.*

Additional Figure 6 Sympathetic ganglia (SG) imaging of sympathetic neuron distribution segments in Group 3: Flexor digitorum profundus (upper), flexor digitorum superficialis, and palmaris longus.

***Additional Figure 7:*** *Sympathetic ganglia (SG) imaging of sympathetic neuron distribution segments in Group 4: Extensor digitorum communis, extensor indicis, and extensor digiti minimi.*

Additional Figure 7 Sympathetic ganglia (SG) imaging of sympathetic neuron distribution segments in Group 4: Extensor digitorum communis, extensor indicis, and extensor digiti minimi.

***Additional Figure 8:*** *Sympathetic ganglia (SG) imaging of sympathetic neuron distribution segments in Group 7: Rectus femoris, vastus medialis, and vastus lateralis.*

Additional Figure 8 Sympathetic ganglia (SG) imaging of sympathetic neuron distribution segments in Group 7: Rectus femoris, vastus medialis, and vastus lateralis.

***Additional Figure 9:*** *Sympathetic ganglia (SG) imaging of sympathetic neuron distribution segments in Group 8: Iliopsoas, gluteus maximus, and pectineus.*

Additional Figure 9 Sympathetic ganglia (SG) imaging of sympathetic neuron distribution segments in Group 8: Iliopsoas, gluteus maximus, and pectineus.

***Additional Figure 10:*** *Sympathetic ganglia (SG) imaging of sympathetic neuron distribution segments in Group 9: Plantaris, tibialis posterior, and flexor digitorum profundus (lower).*

Additional Figure 10 Sympathetic ganglia (SG) imaging of sympathetic neuron distribution segments in Group 9: Plantaris, tibialis posterior, and flexor digitorum profundus (lower).

## Data Availability

*All relevant data are within the paper and its Additional files*.
