# Supplementary figures and images for: Inductive analysis of the spatial distribution characteristics of neurons that innervate skeletal muscle and their correlation with muscle phenotype
Source: Neural Regen Res. 2025 Aug 13;21(6):2669–80. doi: 10.4103/NRR.NRR-D-24-01540 (PMC13217378; doi:10.4103/NRR.NRR-D-24-01540)

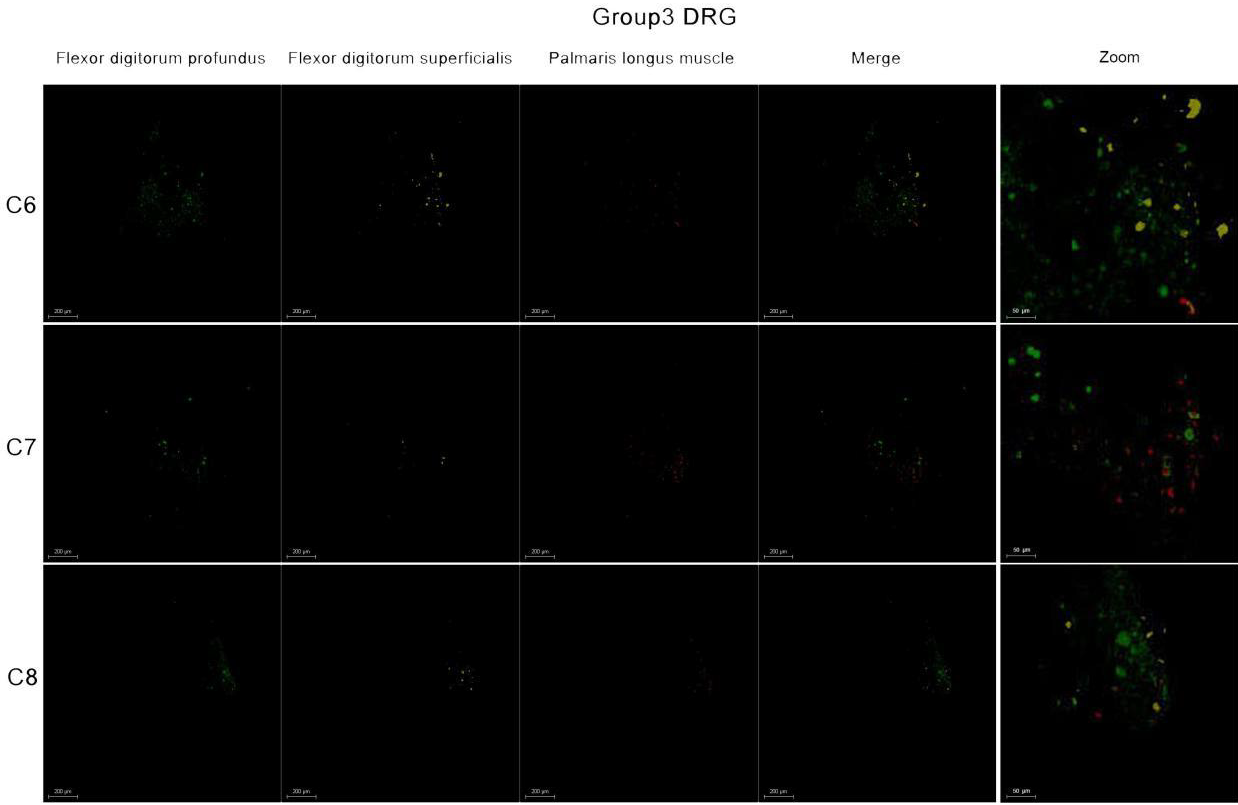

Supplement: Supplementary file 1 [file NRR-21-2669_Suppl1.tif]

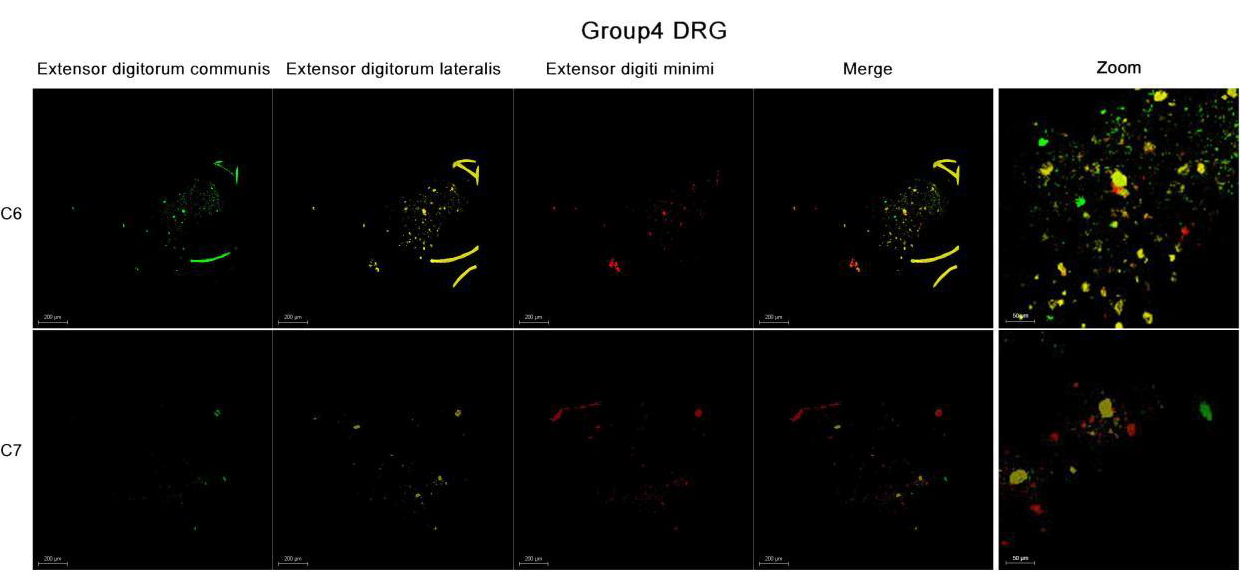

Supplement: Supplementary file 2 [file NRR-21-2669_Suppl2.tif]

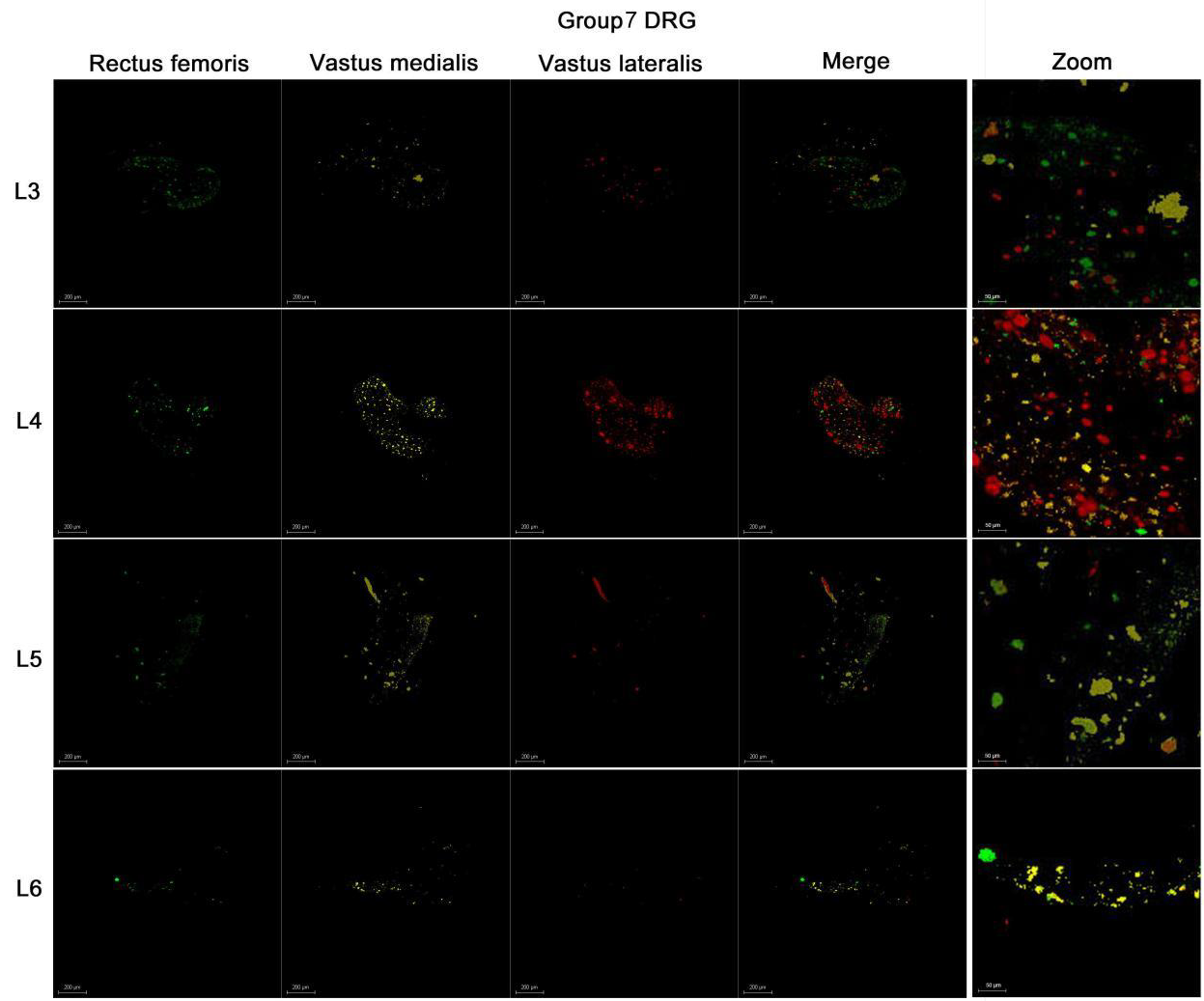

Supplement: Supplementary file 3 [file NRR-21-2669_Suppl3.tif]

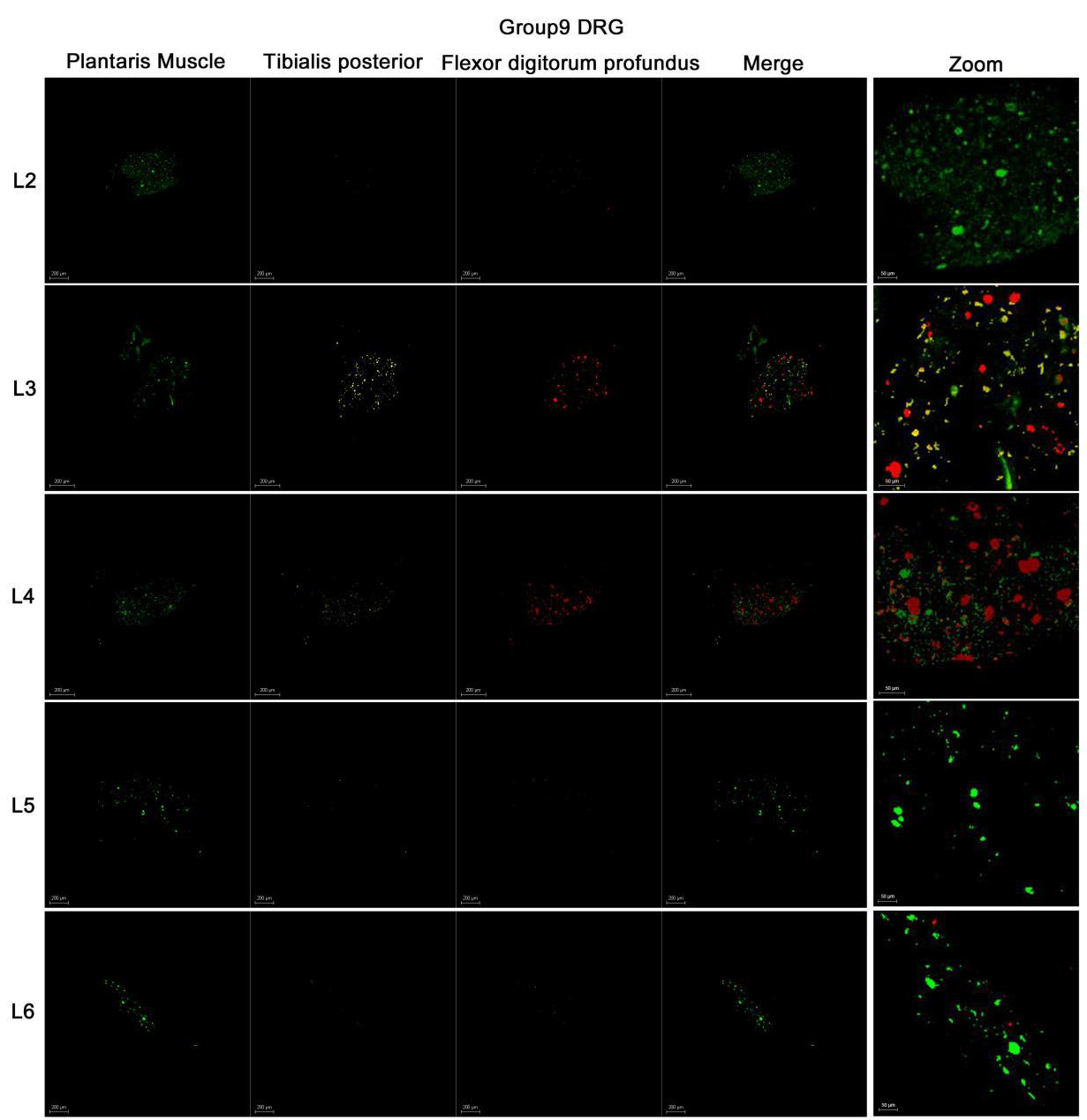

Supplement: Supplementary file 4 [file NRR-21-2669_Suppl5.tif]

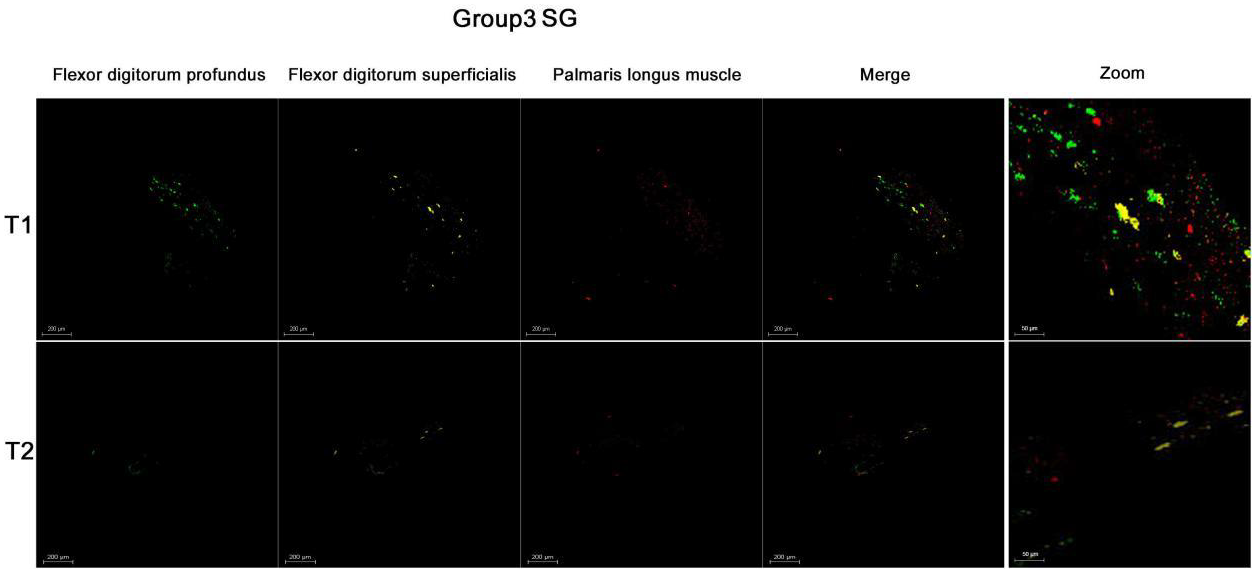

Supplement: Supplementary file 5 [file NRR-21-2669_Suppl6.tif]

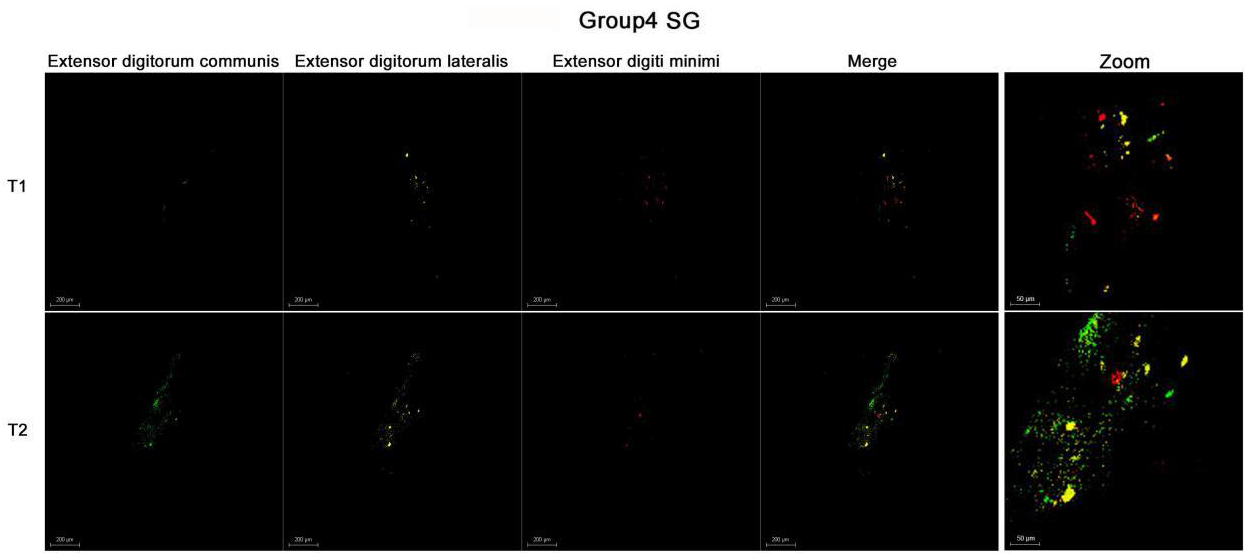

Supplement: Supplementary file 6 [file NRR-21-2669_Suppl7.tif]

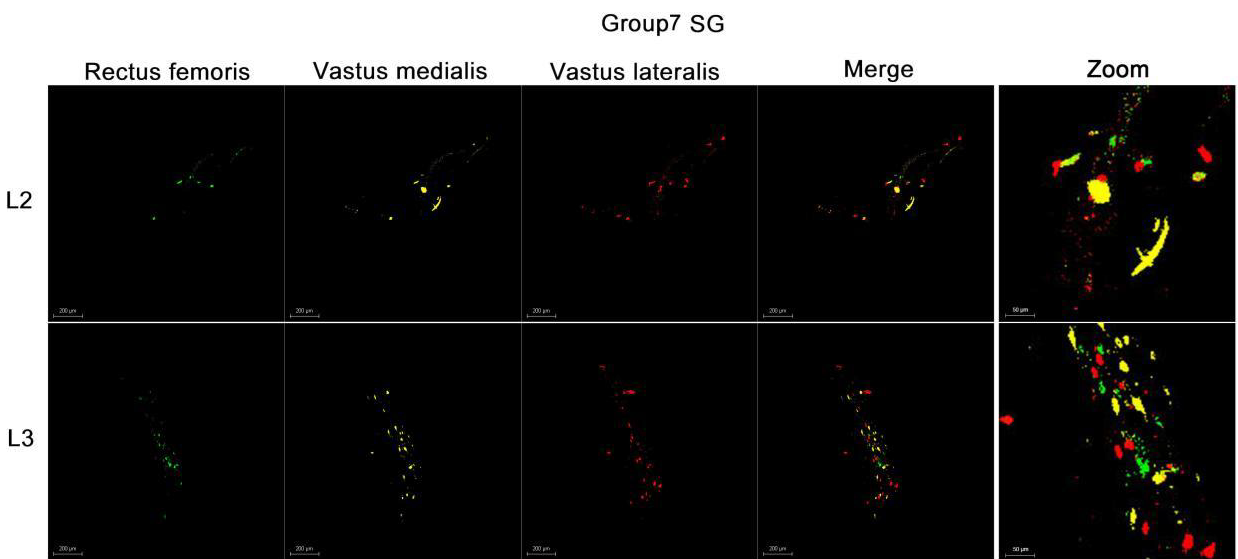

Supplement: Supplementary file 7 [file NRR-21-2669_Suppl8.tif]

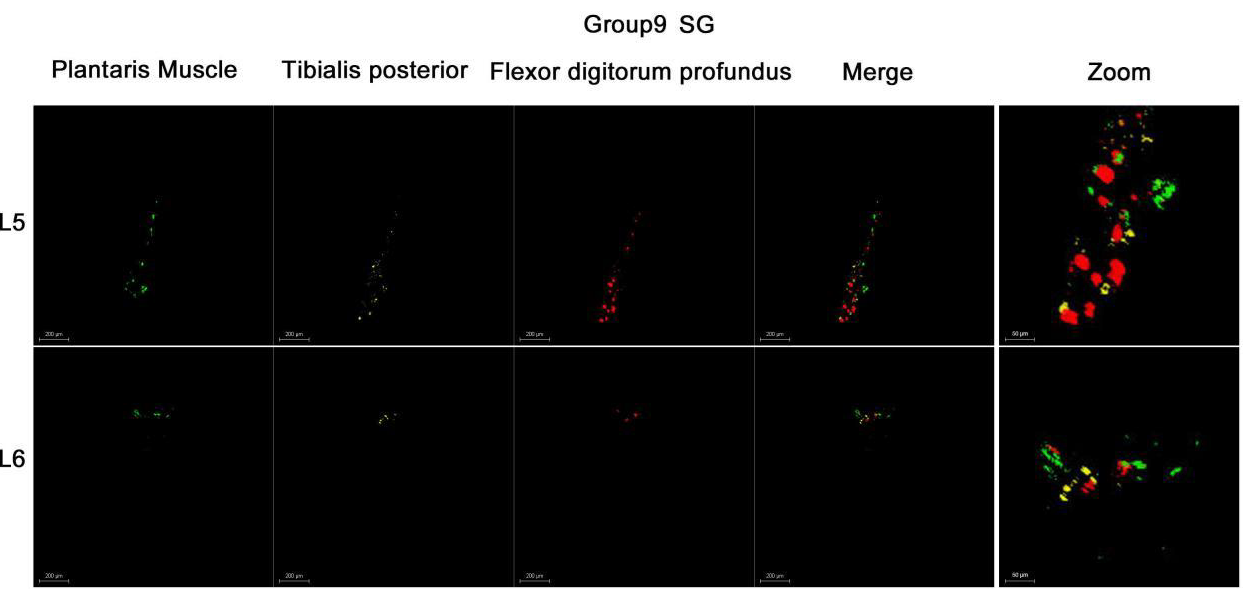

Supplement: Supplementary file 8 [file NRR-21-2669_Suppl10.tif]

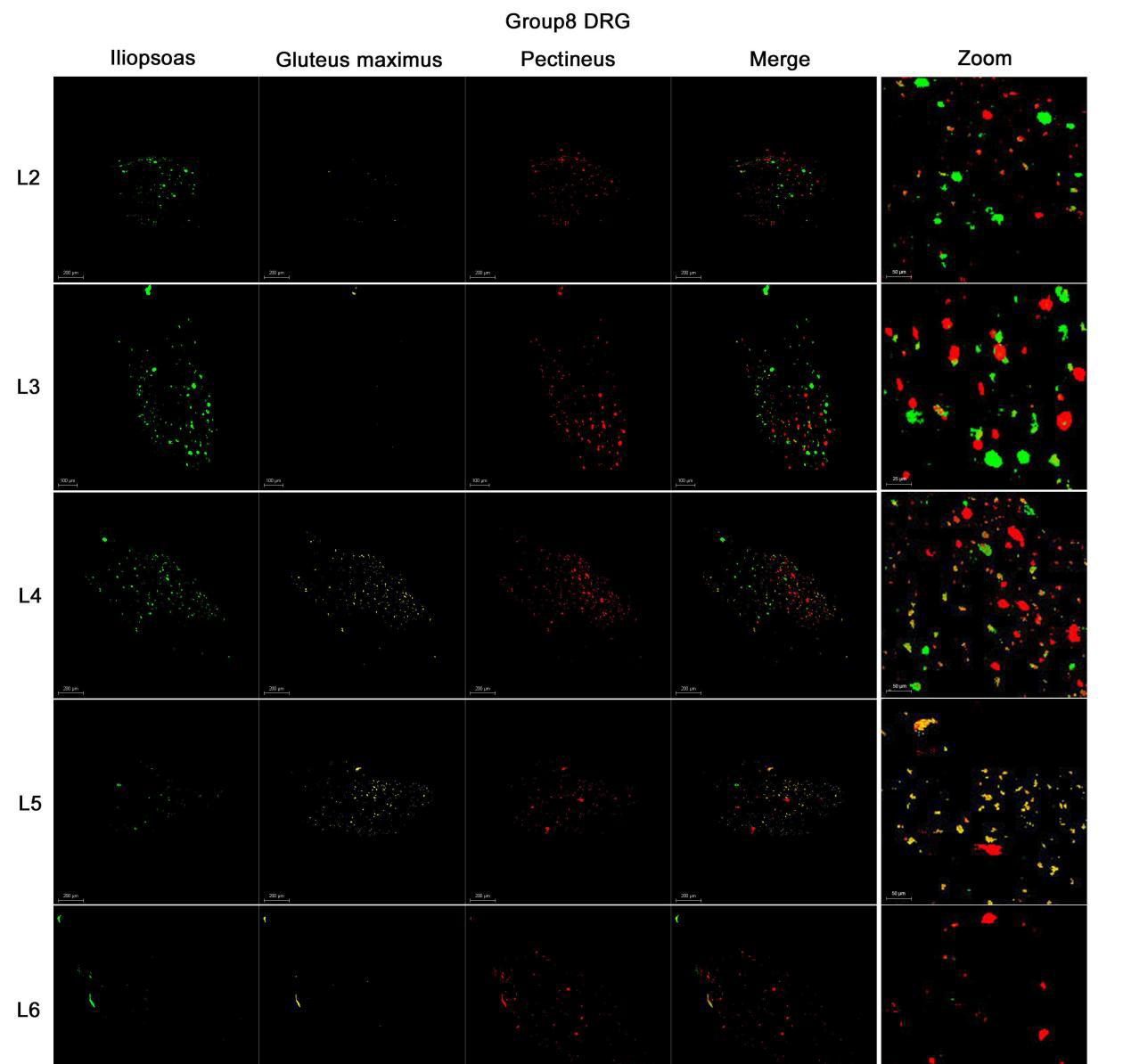

Supplement: Supplementary file 10 [file NRR-21-2669_Suppl4.tif]

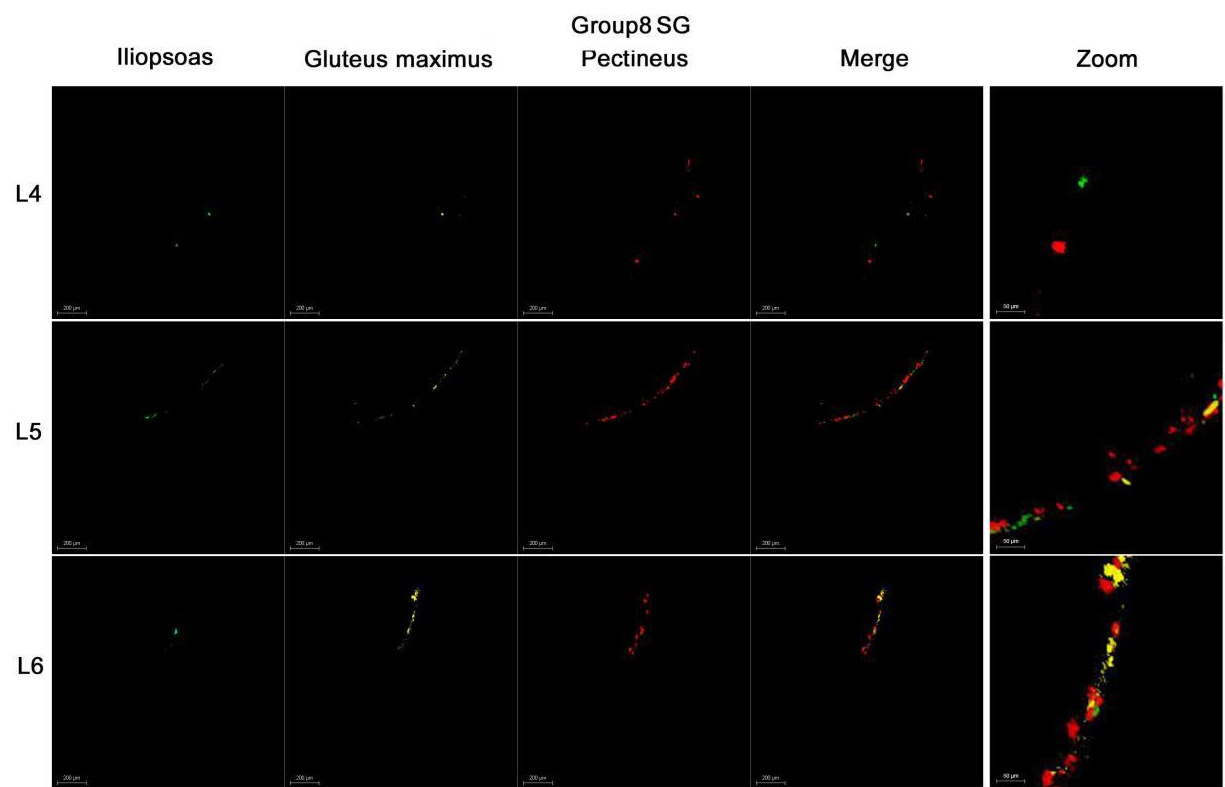

Supplement: Supplementary file 11 [file NRR-21-2669_Suppl9.tif]
