# Supplementary material for: Inductive analysis of the spatial distribution characteristics of neurons that innervate skeletal muscle and their correlation with muscle phenotype
Source: Neural Regen Res. 2025 Aug 13;21(6):2669–80. doi: 10.4103/NRR.NRR-D-24-01540 (PMC13217378; doi:10.4103/NRR.NRR-D-24-01540)
Supplement: Supplementary file 9 [file NRR-21-2669_Suppl1.pdf]

Additional Table 4 List of neuron-related genes

Sensory neuron positive related genes	Sensory neuron negative related genes	Sympathetic neuron positive related genes	Sympathetic neuron negative related genes	Motor neuron positive related genes	Motor neuron negative related genes
Ascc2	Tsc22d3	Akap12	Rph3al	Ecm2	Xrcc1
Egln3	Ubac1	Sulf1	Peli2	Lhfpl2	Mrps24
Megf9	Clec10a	Kctd12b	Fam189b	Serinc5	Slc25a28
Wnt5b	Rfx5	Lama4	Gmpr	Ankrd29	Cisd1
Eif1ad	Ifi47	Lima1	Syngr1	Scrn1	Sdhaf1
Casp9	Ndel1	Dio2	Ptov1	Rab31	Ubac2
Nudt13	6430571L13Rik	Ptgfrn	Adi1	Aldh18a1	Rnf166
Gstm7	Pfkfb4	Arl4c	Abhd11	Specc1	Cobl
Atp1b4	Psmd8	Thbs3	Cisd1	Pamr1	Cds1
Zfp346	Pfkm	Atrnl1	Dnajb12	Cnn3	Dnajb12
Kcnn3	Exoc7	Cp	Ubac2	Slc36a4	Ndufa7
Zfp446	Ggt5	Bub1b	Stradb	Ednrb	Ddr1
Napepld	Lin9	Dock1	Myod1	Alox5	Mrpl16
Acss1	Cgrrf1	Nrcam	Romo1	Igsf10	Tfcp2l1
Dclk3	Hilpda	Gas7	Ogg1	Scara3	Tnip1
Acot2	Gbp5	Col8a2	Atp6v1f	Clec11a	Mier2
Atp2a2	Farsa	Lhfpl2	Mrps24	Lhfp	Hps4
Hspa1b	Rilpl2	Clec11a	Metrn	Pdgfrl	Dlg4
Fam120c	Marcksl1	Tmod3	Nle1	Gfpt2	Poll
Palmd	Mrps28	Vcan	Eif4e3	Lima1	Atp5g3
Sema3f	Triobp	Col11a1	Taz	Lama3	Fam98c
Med14	Slc11a2	Crispld1	Noc4l	Nrn1	Scly
Fzd1	Jmjd6	Ptx4	Pla2g6	Pls3	Rhobtb2
Ldlrad3	Sgk1	Pafah1b2	Dexi	Man1a	Cux1
Dok5	Ankrd9	Myof	Mrpl37	Fat1	Rabac1
Stau2	Dupd1	Stard13	Fitm1	Apold1	Pde4a
Aldh7a1	Frat2	Ptpn13	Gpaa1	Vav1	Ldb3
Fzd6	Nans	Slc39a10	Ndufs7	Svep1	Mgat4b
Drp2	Rpia	Zfp185	Spns2	Nedd9	Peli2
Gnb1l	Bet1	Prickle2	Atxn7l2	Ptpro	Slc2a8
Ercc3	Chchd1	Zfp110	Xrcc1	Dio2	Spns2
Vps26b	Nudt19	Col14a1	Metap1d	Itga11	Tusc2
Fbxl2	Unc93b1	Tubb2b	Rnf126	Duox1	Sys1
Use1	Pdlim7	Fstl1	Cpped1	Slc41a2	Mrpl45
Rims4	Abcg2	Tbc1d9	Parp6	Aspn	Creld1
Plxna4	Slc35e4	Cttnbp2nl	Hps4	Thbs3	Srebf1
Nup188	Tpm1	Alox5	Dusp23	Cdh11	C8g
Plbd1	Polr2i	Cfh	Mknk1	Il13ra1	Dctn2
Scube2	Mylpf	Arhgap29	Hspbp1	Edem1	Rtkn
Eftud2	Ift122	Man2b1	Samd10	Mkx	Blcap
Kctd1	Pop5	Srgap3	Rapsn	Dse	Metap1d
F2rl1	Pigc	Olfml2a	Ndufb6	Armcx1	Fam122a
Ppara	Tprkb	Ext1	Alg1	Atp10a	Dcaf6
Myrip	Ier3ip1	Adcy7	Mamstr	Adamts2	Rhbdd3
Thsd1	Ankrd24	Loxl2	Rnf166	Gas7	Prkaca
Prss12	Hint1	Glce	Zdhhc16	Tbl2	B4galt3
Cobll1	Tmem81	Galnt4	Lrrc20	Tmtc2	Sugp1
Slc7a1	Ccdc34	Nup107	Prr13	Cpxm2	Pias4
Gab2	Tmem100	Manba	Ddx49	Tbc1d2b	1700001O22Rik
Slc7a5	Arl16	Fat1	Cars2	Itgbl1	Mapk14
Lrrn1	Ubxn1	Nrp1	Top1mt	Cyth3	Atp6v0b
Zfp827	Pak1ip1	Sf3b3	Asb8	Arl4c	Rnf8
Trib3	Stk38	Tmem168	Ube2f	Pcsk6	Anapc11

Lhfpl4	Sfr1	Cd109	2210016L21Rik	Sec24d	Hlf
Pdk4	Zfp326	Chodl	Sdhaf1	Nrcam	Daxx
Iars	Exosc7	Ifit2	Ypel3	Sparcl1	Metrn
Il6ra	Dusp3	Fam3c	Slc25a39	Emp1	Ptov1
Icam1	Gimap9	Itga8	Smyd2	Syn2	Gja4
Usp46	Pgam2	Atp6ap2	Gmeb2	Zfp658	Acot8
Fem1c	Renbp	Ankfy1	Ppif	Spats2l	Mrpl14
Slc9a4	Ndufs6	Samd9l	Rps6kb2	Sema3b	Aip
Ss18l1	Calm2	Adamts6	Atp5d	9930111J21Rik1	Dok7
Tmie	Cdc42ep3	Ednrb	Arrb2	Dock11	Aamp
Fgf2	Tnfaip2	Btnl9	4833439L19Rik	Lox	Nle1
Tmem131	Clns1a	Camk4	B4galt3	Col13a1	Nudt6
Astn2	Cwc25	Ppm1e	Ndufa3	Enpep	Romo1
Dact3	A430005L14Rik	Mcm6	Pcid2	Bmp3	Lrrc20
Fam13a	Suclg1	Cmtm6	Itfg2	Sulf1	Smad3
Coq3	F8a	Frk	Ost4	Ubxn2b	Bola3
Acaa2	Hps5	Brca2	Rfng	Tmed8	Pgam5
Man1c1	H2-T22	Maff	Porcn	Lamp2	Sav1
Tnni1	Tmem37	Dock10	Cops7b	Stard13	Prr13
Tnnt2	Tmem101	Med17	Banf1	Col8a1	Fastkd1
Lclat1	Pfdn1	Plce1	Nup210	Ugdh	Pebp1
Tbx3	Mpp3	Adamtsl3	E2f4	Arfgap3	Myod1
Lix1l	Ddx41	Pdgfrl	Mical2	Tbc1d9	Pla2g6
Fam185a	Katnal2	Pcdh12	Rab20	Fn1	Srrd
Dusp18	Nedd8	Pam	Naa10	Olfml2b	Neurl1a
D630045J12Rik	Nmb	Tpp2	Gps2	Rasa3	Josd2
Gramd1b	Tnni2	Rcn1	Vgll4	Ogn	Fgf13
Gabarapl1	Serpinb6a	Matn2	Lrg1	Vcan	Abcb9
Sv2a	Rpl22l1	Mob1a	Tpra1	Col8a2	Psmd3
Ints7	B4galt7	Col16a1	Shisa4	Adamts16	Pkdcc
Fzd9	Emg1	Abhd2	Ubl5	Adcy7	Atp6v1f
Zfp770	Psmb6	Lrrc8d	Map1lc3b	Ntn1	Actr3b
Rfx2	Cd320	Vav1	Pex11b	Grb10	Fam76a
Dapp1	Rom1	Dsel	Cuta	Tmem119	Zdhhc16
Tbx4	Zmym1	Gnai3	Scly	Prps2	Det1
Fam117b	Tmem199	Fgl2	Cox6a2	Sf3b3	Tcea3
Btg1	Uqcr10	Parn	Nubp1	Sqle	Pih1d1
Ostn	Mrpl13	Nrn1	Rnf5	Necab1	Map1lc3a
Sh3bp4	Mpnd	Dock11	Lsm12	Oaf	Cpsf4
Pfn4	Tia1	Msh3	Grina	Pcdh12	Hcfc1r1
Lgi1	Mettl17	Apaf1	Snx3	Kctd12b	Dazap1
Kif1a	Pag1	Utp20	Zmat5	Itga8	Tmem11
Ttc9	Mrpl42	Igsf10	Taf1c	Pbld2	Cish
Dbn1	Kcnj12	Chml	Tmem160	Pcdh18	Map3k3
Srgap1	Prpf3	Rab31	Arfgap1	Fat4	Rfng
Tab3	Thap4	Cgnl1	Zfand3	Ppm1e	Dcaf15
Taf2	Gtf2a2	Col8a1	Arl2	Antxr2	Lbx1
Wdr41	Eif3i	Man1a	Sav1	Prkci	Gemin7
Osbp2	Abcc5	Naip2	Ube2l3	H3f3b	Slc25a3
Syt9	Anapc5	Rftn2	Csnk2b	Col11a1	Rab3a
Cpsf3	Pigp	Aldh18a1	Mbd1	Spin4	Irak1
Esrrg	Ndufa6	Endod1	Ddx19a	Rassf2	Cars2
Kif7	Mgst3	Col22a1	Ube2d1	Myo1e	Ddx19a
Tpm3	Fbxl4	Afap1	Ppm1j	Prtg	Gmpr
Hadh	Dut	Nedd9	Dnajc30	Chrdl1	Rph3al
Hspa1a	Pik3ip1	Kif13b	Zfp358	Loxl4	Trim28
Abtb2	Zfp467	Tigd4	Fhl3	Pcsk5	Ppp2r5b

Ckb	Stbd1	Acap2	2310061I04Rik	Cd83	Wdr24
Stc1	Zbtb8os	Pole	Kctd13	Sec16b	Hdgf
Ppp1r2	Drap1	Pros1	Rad23a	Dock10	Sctr
Gtf3c1	Polb	Topbp1	E430018J23Rik	Ptpn13	Trim8
Ube2z	Gadd45g	Atl3	Aup1	Zfp110	Zswim1
Myh7	Dkk3	Gnb4	Znhit1	Rcn1	Nckipsd
Gtpbp1	Fahd1	Epha3	Fis1	Ptx4	Ost4
Efr3b	Ctnnbip1	Ppip5k2	Vps72	AW549877	Rad23a
Ampd3	Ccdc91	Plekhg1	Ppt2	Akap5	Arid3b
Pcp4l1	Atp6v1e1	Syn2	Mrpl4	Lgr5	Fam189b
Pik3r4	Ccdc137	Mgat5	Tmem150a	Tnc	Pnmt
Abat	Ttc1	Cdk17	Lrrc38	Pafah1b2	Sesn2
Kbtbd11	Znhit3	Atp8b1	Agpat1	Kcnk5	Rapsn
Hpdl	Ndufb8	Serinc5	9130401M01Rik	Aldh1a3	Eif4e3
Mapk6	Chrac1	Prrg3	Nudt6	Ptgis	Dhps
Stk24	Rnf121	Ckap5	Sypl2	Tnfrsf22	Smad1
Cyfip2	Mcts1	Mmrn2	Fastk	Ncf2	Chchd10
Foxo3	Sh3kbp1	Nckap1l	Serinc2	Plxnc1	Smarcd3
Hibadh	Sf3b5	Pde5a	Abcb9	Cd109	Bud13
Tceal3	Tmsb4x	Spats2l	Ncdn	Col14a1	Fiz1
Rsl1	Ndufa2	Ripk1	Tecr	Map4k3	Snx3
Tmc7	Calm3	Dpp4	Atp1b2	Ppip5k2	Tob2
Pik3cb	Glmn	Lrrk2	Atp6v0b	Zfp619	Coro7
Klhl36	Ccdc28b	H3f3b	Rnf167	Bub1b	Zer1
Heg1	Ntf5	Svep1	Dtx2	Meox1	Atxn7l2
Emp2	Rabepk	Rnf19a	Clpp	Vps4b	Gmeb2
Tnnc1	Psma6	Osbpl8	Tcea3	Ust	Gpc1
Dtx4	Slc25a14	Zfp518a	Tex261	Atrnl1	Glrx5
Grsf1	Gpn3	Snx4	Dusp10	Cdr2	Adck2
Chrnd	Dolk	Dpysl3	Fam76a	Pik3cg	Vps72
Fgr	Med8	Slc36a4	Ap1s1	Sgpl1	Abhd11
Inpp5j	Thap11	Ntn1	Fam98c	Lama4	Map2k7
Arhgap42	Rnf130	Tbc1d8b	Sec61a2	Prickle2	2310061I04Rik
Kdm3b	Hoxa10	Strn	Map2k3	Tgfbi	Ccm2
Lancl3	Id3	Rnd1	C8g	Dnm1l	Arf5
Decr2	Kcng4	Lepr	Wbp2	Pygb	Taf10
Myom3	Myom2	Slc30a5	Sys1	Anxa8	Paqr7
Fbxo21	Srp14	Arhgef3	Tmem175	Rab27b	Zcchc24
Galns	Tapbpl	Tspan2	Plscr3	Acap2	Cnksr1
Rinl	Snx24	Pdgfd	Rabac1	Wif1	Eif1
Stmn2	Sdf2	Cd93	Eif1	Naip2	Sdhc
Ranbp9	Mrpl54	Frem1	Csnk1e	Ncf1	Pygo2
Prepl	Nhp2	Syde2	1110065P20Rik	Mthfd1	Mafg
Wdr1	Ccnl1	Zfp810	Gnb5	Cdh2	Usf2
Gpkow	Mrpl20	Cplx2	Sharpin	Zfp948	Ptpmt1
Tal1	Srsf11	Csf1r	Rnf123	Nckap1l	Syngr1
Smo	Zfp94	Grb10	Ppp3cb	Cttnbp2nl	Il34
Nsun3	Pik3c3	Emb	Sdr39u1	Crispld1	Ubqln4
Dhcr24	Sec11c	Rassf2	Bcl6	Manba	Lrrc38
Ppp1r3d	Ddx47	Sema3d	Scamp3	Zfp185	Rab20
Gca	Zfp112	Dock7	Reep5	Btnl9	Zdhhc8
Acss2	Nudcd2	Tlr4	Zdhhc8	Asap3	Pias3
Pik3r5	Rbak	Cenpc1	Slc25a3	Bfsp1	Fbxl12
Tmem47	Tnfaip3	Morc3	Gpx3	Cpt1a	Osgin1
Sh3rf2	Fam131a	Pibf1	Dhps	Afap1	Fhl3
Clptm1	Ttc4	Rbbp8	Dlg4	Tek	Ppm1j
Antxr1	Thap3	Scrn1	Plekhb1	Fndc3a	Neurl2

Inhba	Fam118b	Kif11	Car11	Asah1	Dtx2
Rtn4ip1	Txndc11	Smyd4	Rusc1	Eif5a2	Rpap1
Zbtb8a	Bub3	Chic1	Carm1	Sema3d	1110008P14Rik
Hivep1	Tmem208	Cenpf	Steap3	Trpm3	Ttc39c
Btrc	Smarcd2	Adamts15	Arf5	Adamtsl2	Cox5a
Lrrc39	Uqcr11	St8sia4	Bop1	Sp3	Map2k3
Pdgfc	Ndufv3	Klhl6	Gorasp2	Plekhg1	Dpp9
Sord	Ccdc28a	Omd	Pdk2	Vsig4	Tmub1
Ptpn12	Srpx	Gem	Fam3a	Atl3	Reep5
Zxdb	Rps21	Edem1	Dagla	Cp	AU040320
Tm4sf1	Olfr78	Gm5431	Mlf2	Slfn2	Tmem9
Pank2	Pctp	Ercc5	Tmem106c	Dclre1a	Phb2
Col9a2	Bpgm	Axl	Rnaseh2c	Kbtbd7	Bcl6
Bcr	Rnf113a1	Mpeg1	Pgam5	Garnl3	Lyrm7
Atg10	Dusp12	Adamts2	Hsf1	Cdon	Cryab
Aida	Fdxacb1	Magi1	Cdk2ap2	Hmgcr	Plekhh3
Rmi1	Mllt11	Mcm4	Vegfb	Usp33	Hspb6
Inpp4a	Luc7l	Slc30a1	Paqr7	Ripk1	Mocs3
Erbb3	Prkrip1	Pcdh18	Rab10	Prcp	Noc4l
Ahr	Osgep	Slc5a3	Txn2	Camk4	Zfand3
Reln	Pfdn5	Akap5	Akr1e1	Camsap2	Fbxw9
Socs5	Thoc7	Ankrd29	Ddr1	Colec12	Epn1
Nup98	Tph1	Ntn4	Lypla2	Plcg2	Tjap1
Dbt	1110004F10Rik	Pamr1	Creld1	Arhgap29	Taz
Wnt16	Tmem60	Ncapg2	Usp10	Vit	Alg1
Enc1	Snrpb	Plxnc1	Coro7	Man2b1	4833439L19Rik
Rybp	Magohb	Lamp2	Scamp2	Bzw1	Ces1d
Polr1b	Zfand6	Yes1	Gemin7	Tmod3	Klf13
Gcnt2	Flywch1	Cd200r3	Smarcd1	Adamts20	Lamp1
Brpf1	Zfp2	Serpinb8	Pias3	Tmem164	Sypl2
Homer2	Ccdc57	Tmtc2	Mif4gd	Tubb2b	Pkig
Asap2	Psma7	Armcx1	Ccdc6	Pdgfd	Mrpl37
Ptprf	Mrps12	Slc41a2	Ctdnep1	Nrp1	Cdc34
Xlr4a	Evi2a	Prkdc	B3galt4	Cybrd1	Ppp1r16a
Rfk	Ficd	Ube3c	Sox7	Spon2	Stradb
Nrd1	Ndufa1	Clec1a	Kcnc4	Dock7	Rhot2
Kcnj3	Mfsd3	Il2	Slc2a8	Syde2	Echdc3
Prep	Hjurp	Aspn	Rangrf	Ifit1	Rab40c
Atf6	Tctex1d2	Fat4	Med25	Pam	Sf3b4
Samm50	Rpain	Dpy19l1	Ywhae	C1qtnf3	Zfp598
Nuak1	Rassf1	Rnd3	Me1	Snx4	Lsm2
Spef1	Htra1	Ranbp6	Pex14	Slc30a7	Coil
Wdr59	Pigw	Gimap8	Tada3	Efcab5	Pex26
Dact1	Cox7a2	Cyp2j6	Cox5a	Sh3bgrl	Lancl1
Robo2	Ppie	Abcb1a	Pknox2	Tpm4	Rnf123
Mrm1	Cnrip1	Ubxn2b	Syvn1	Cx3cr1	Acd
Hccs	Dhodh	Cyp51	Rilp	Mgat5	Tbc1d10b
St6galnac4	Vmp1	Klhl4	Mon1a	Calcrl	Tmbim6
Zfp423	Hscb	Nin	Mrpl16	Myof	Ncdn
Nploc4	Chka	Meox1	Pde4a	Exoc6	Itfg2
Adamts14	Mitd1	Dstyk	Ldb3	Enpp1	Pdk2
Ap1ar	Psmd6	Srpx2	Mpv17l2	Notch2	Scn1b
Ttc38	Mrps17	Enpep	Trmt112	Thbs2	Rasa4
Mamdc2	Napg	Sema3b	B3gat3	Picalm	Brf2
Nt5c2	Rplp2	Pign	Osgin1	Sec61a1	Slc25a39
Wdr48	Gsdmd	Prex1	Map2k7	Megf10	Ddx49
Syt17	Per3	Vsig4	Rtkn	Tlr13	Pnpla2

Alx4	Mlf1	Top2a	Fgf13	Dock1	Thtpa
Col23a1	Ctr9	Nes	Cobl	Emilin2	Fastk
Pdlim1	Rad1	Ubash3b	Phc1	Ggta1	2210016L21Rik
Maml2	Fxn	Pi4k2b	Rab3a	Zmym6	Tex261
Rbm15b	Clec4a2	Acsl5	Copz1	Atrn	Tfeb
Rasl11b	Ndufb2	Abca1	G6pc3	Ercc5	Dexi
Itpr3	Hsd11b1	Zfyve16	Sdhc	Chic1	Guk1
Mvd	Ndufa5	Gcnt1	Klhdc3	Trpc6	Snrpn
Pde7b	Cys1	Mmp16	Adrm1	Ctsl	Cpped1
Slit3	Nt5c3	Copb2	Syt3	Wdr82	Rnf157
Cnnm2	Pde4b	Cep192	Slc25a44	Kif13b	Tbc1d17
Ahcyl2	Zfp61	Abca5	Lancl1	Os9	Adpgk
Xpo4	Rps14	Avl9	Smarcd3	Rab8b	C2cd2l
Arhgap32	Gjc1	Cnn3	Tmem222	Col16a1	Snx12
Endou	Mrpl32	Kcnk5	Hspb6	Tubb5	Mboat7
Mid2	Rhebl1	Pcdhb16	Taf6	Polr3b	Asb16
Zfp746	Tmem69	Slamf1	Max	Ext1	Golph3
Dnajc14	Gpx7	Zfp820	Gsk3a	Nid1	Rasd2
Gab1	Zc3hc1	Cd83	Fam122a	Dpp4	Cc2d1a
Tmem64	Mtg1	Dse	Tpp1	Ptk7	Klc2
Rassf8	Spr	Has2	Gnas	Cilp2	Rnf187
P2rx1	Fdx1	Pdcl	Polr3c	Manea	Lypla2
Myo18b	Polr3g	Atad2	Tmem203	Ipo11	Dusp23
Pvr	Cr1l	App	Dph2	Rnf19a	Ptpn1
Lmod2	Ddx28	Plcb1	St8sia5	Nckap1	Ntpcr
Hdac9	Mrpl21	Notch2	Camk2g	Cmtm6	Gnas
Src	Psmd13	Kbtbd2	Zfpl1	Brca2	Asb8
Prkaa1	Aimp1	Nup205	Plekhj1	Gulp1	Efna1
Ror2	Pfdn4	Scg3	Ascc1	Akap12	Rab10
2200002D01Rik	Zfp263	Osbpl3	Ipo13	Rgs6	Ogg1
Rnf144b	Mxd4	Specc1	Ighmbp2	Adamts6	Dennd4b
Chsy1	Map2k1	Tnc	Phpt1	Gas2l3	Rhbdl3
Asxl1	Zfp58	Zfp790	Epn1	Tbc1d12	Ppt2
Zc3h12c	Cbfb	Parp14	Zdhhc18	Lrrn4cl	Naa10
Mex3b	Dohh	Pdgfra	Men1	Ankib1	Riiad1
Sertad4	Tmed3	Pcsk5	Tmem80	Otud6b	Sh3bp1
Mcc	Kcnab1	Pik3cg	Tesk1	Rnf2	Wbp2
Sdk1	Rbm17	Dock8	Pnpla2	Smyd4	Ndufa3
Ddx1	Amz2	Pi15	Cish	Abra	Rnf167
Ptpn14	Ccdc146	Npat	Taf10	Pdgfra	Comtd1
Slc9a2	S100a4	AW551984	Podn	Gcnt1	Traf3ip2
Fbln1	Wdr53	Flnb	Comtd1	Bche	Pex14
Cyyr1	Gng11	Lims1	Tuba8	Arhgef3	Sgsm3
Tecta	Magoh	Fbxo30	Erp44	Scg3	Stub1
Llgl2	Cdkn2d	Anxa4	Cdk20	Yeats2	Mta1
Oxsm	Med4	Plxnb2	Slc25a28	Hmcn1	Thyn1
Ltbp2	Spc24	Fbn1	Lbx1	Edem3	Hoxb4
Cdv3	Fbp2	Adam10	Pias4	Mtx3	Rab12
Aif1l	Ppa2	Acvrl1	Rnpep	Atp8b1	Hspb2
Mrrf	Mrpl33	Pcsk6	Bcl7b	Lrrc8d	Cuedc1
Znrf2	Smarca1	Epha4	Timp4	Tmem167b	Ndufb6
Imp4	Ptp4a2	Kbtbd7	Nr1h2	BC034090	Kcnj11
Myh7b	Cnot8	Msr1	Dennd4b	Mmp2	Nfatc2ip
Ppp1r26	Aif1	Emilin2	Atxn7l1	Arhgef10	Nsun4
Anks1	Tmem67	Ecm2	Chchd7	Fat3	Hoxa7
Mpzl1	Ppp1r11	Arfgap3	Hdgf	Slc35f1	Bag6
2310057M21Rik	Stat6	Sqle	Rbm38	Rnf115	Ctdnep1

Slc9a7	Cables2	Plod2	Dalrd3	Hif1a	Cox6a2
Tfcp2	Ube2e1	Anp32a	Snrpc	Uqcrc2	Erp44
Ptpdc1	Skap2	Xpo1	Sirt2	Omd	Vegfb
Fut11	Elof1	Pabpc4l	Fam78a	Mmp16	Fance
Atxn10	Arhgap15	Atp11c	Hr	Itgb2	Kctd15
Itgb4	Bphl	Prex2	Txnl4b	Klhl32	Rusc1
BC048679	Eif4e	Lama2	Gypc	Matn2	Slc30a2
Fxyd6	Tpx2	Lama3	Mapre3	Prkaa1	Tsc22d4
Fgfr3	Mrpl22	Hmgcr	Mrpl55	Ddx1	Ets2
Asah2	Art1	Enpp2	Dpf2	Tmem168	Tnk2
Nacad	Dctn3	Cog3	Sf3b4	Mertk	Adi1
Tmem132a	Naa16	Adam22	1110008P14Rik	Frem1	Smyd2
Tuba1c	Pnkp	Ptprr	Entpd6	Nup107	B4galt5
Paqr8	Atp5k	Lrch2	Dda1	Tmtc3	Tmem80
Klhl3	Asph	Srbd1	Pold2	Gimap8	Rapgef1
Sh3bgrl2	Slc2a3	Ptprc	Six5	App	Tmem106c
Ccdc63	Slc25a19	Camsap2	3110082I17Rik	Srgap3	Fbxo46
Stx11	Polr1d	Sgk3	Bag6	Slc39a10	Tmem52
Lrrc49	Abhd16a	Zfp677	Ccnk	Slc2a13	Tecr
Dffa	Cd55	Rab27b	Deaf1	Il10ra	Acvr2b
Slco2a1	Palb2	Fam114a1	Rpusd3	Col12a1	Suv39h1
Lrrc8b	Bex1	Slfn2	Efna1	Uba3	Acads
Myo1d	Reep1	Megf10	Dym	Pdcl	Mrps34
Nob1	Tor1b	Pla2g4a	Rnf187	Gem	Ncln
Ccdc85a	Tnnt3	Fndc1	B4galt5	Fndc4	Mrps23
Scaf8	Ormdl1	Abca9	Dysf	Ptprd	E430018J23Rik
Suclg2	2610001J05Rik	Scara5	Mafb	Dnmt1	Hr
Trim13	Tspan8	Trappc8	Nop2	AW551984	Gpaa1
Dock5	Mrpl15	Tmed5	Lrrc14b	Aff3	Spry2
Thbs1	Rbx1	Os9	Phlda3	Zfp53	Itpk1
Emp1	Mpst	Angpt1	Crtc2	Has2	Polm
Ankrd35	Eif2b4	Ythdc2	Cryab	Adamtsl3	Arrb2
Myl2	Cryzl1	Aldh1a3	Ndufa7	Slc28a3	1110065P20Rik
Rabl3	Psmb1	Me2	Asb16	Gja1	Psmc4
Prpf8	Mrpl2	9930111J21Rik1	Thap7	Pign	Fth1
Zfp59	Tssc4	Ptn	Atp6v0a1	Ckap5	Tomm40
C1ql3	Ppid	Dok1	Tspan7	Ankrd1	Synpo2l
Dusp16	Tbc1d13	Fbxo38	Dennd1a	Utp20	Dysf
Lrp12	Itgb1bp1	Rngtt	Nrtn	Fbn1	Mamstr
Lamb3	Zfp768	Ikbip	Hsbp1	Antxr1	Mrpl38
Kcns3	Gstt2	Antxr2	Dedd	Sh3bgrl2	Ywhae
Ttll11	Mgl2	Gmfb	Rhobtb2	Gm5431	Smarcd1
Fgf7	Sertad3	Spred1	Tab2	Cd200r3	Cand2
Tnnt1	Srp19	Ccr2	Psme3	Ptn	Rnaseh2c
Zfp36l2	Pomp	Irf4	Mdga1	Abcb7	Nup210
St3gal3	Bccip	Rab33b	Usp2	Il17rd	Tesk1
Ipmk	1600014C10Rik	Galnt5	Ubqln4	Avl9	Smn1
Lman2	Cxcl14	Itgb1	Dyrk1b	Ifit2	Ipo13
Zfp609	Slc35b2	Zfp619	Vamp1	Adamts15	Smtn
Gpc4	Ndufa4	Swap70	Parp16	Copg2	Trak1
Slc17a5	Cd151	Gopc	Ap1m1	Rnf13	Rnf126
Ephb3	Sec61b	Mkx	Tfe3	Morc3	Txnl4b
Msl2	Ppp1r14b	Usp29	Bud13	Fndc1	Mrpl12
Sphk1	Ubxn6	Golim4	Ctsb	Zfp820	Ranbp3
Vps11	Hirip3	Cyth3	Atp5g3	Slc25a32	Phlda3
Gnmt	Snf8	Nckap1	Zfp687	Tceal3	Klhl30
Myot	Akap8l	Vwa5a	Psmc4	Cdc42ep4	Mif4gd

Cep120
Tmem158
Gna12
Rcor2
Tbx2
Got2
Slc35f5
Irf1
P4ha2
Psmd4
Il12a
Caml
Maf1
Higd1b
Tef
Myh4
Blvra
S100a13
Snupn
Ak1
Upf3b
Aga
Dhrs7c
Fancl
Sh3bgr
Glrx3
Acyp1
Tsen2
Taldo1
Rpp21
Sertad1
Mrpl18
Mcm7
Exosc4
Atg12
Rab40b
Mocos
Idh3a
Impa1
Ky
Ms4a6c
Nfyb
Rnf215
Phf23
Agpat4
Sephs2
Dnase1l1
Psmb8
Tmem29
Smoc2
Tm2d2
Rpl14
Lrrc30
D8Ertd738e
Med30
Chchd4
Frmd8
Fbxw17
Dnajc8
2310002L09Rik
Rsrc1
Pde1a
Ppil1
Ttc19
Cpxm2
Sacm1l
Itgbl1
Colec12
Kcnt2
Tnfrsf22
Alms1
Pikfyve
Zfp229
Kif18a
Ugdh
Foxn2
Luzp1
Arhgap28
Zfp101
Garnl3
Nck1
Smc1a
Slc16a1
Cdr2
Tlr7
Mrc1
Trpm3
Efcab5
Vit
Zfp748
Nudcd1
Sec16b
Slc39a6
Tbk1
Exoc1
Abca8b
Pigg
Stag1
Rttn
Anxa6
Atp10a
Kdm1b
Zfp606
Zbtb41
Sp3
Edem3
Zfp953
Ptpro
Polr3b
Zfp449
Lrig2
BC034090
Snx7
Zfp709
Mgat4a
Naalad2
Rgs6
Zfp758
Lrba
Ogn
Il1rap
Mta1
Sil1
1700001O22Rik
Tmbim6
Ntpcr
Pnmt
Kat2a
Mef2d
Atp6v0d1
Nfic
Pofut2
Gtf3c2
Rab1b
Scarb1
Higd2a
Itpk1
Kti12
Brf2
Zcchc24
Dazap2
Ilk
Hgs
Rexo2
Mrpl38
Echdc3
Wnk2
Commd4
Hspb2
Cox10
AI837181
Wdtc1
Wars
Tmem192
Cd74
Tfcp2l1
Prkag2
Usp14
Morn4
Pigs
Mapk14
H13
Fth1
Cpsf4
Il34
Eif3f
R3hdm2
Sppl3
Ppp2r5b
Tmem140
Zfp707
Prkab1
Tmub1
Pgp
E130309D02Rik
Snapc2
Ssbp3
Rab12
Dcp1a
Pdia4
Tuba1c
Greb1l
Dennd4a
Samd9l
Exoc2
Abce1
Rgs17
Pnrc2
Cenpa
Hps3
Slamf1
Chml
Cdk1
F2r
Prkg1
Timp1
Myo7a
Prdm5
Tjp1
Flnb
Srsf3
Abhd13
Ankfy1
Gria3
Lama2
Vps26a
Usp29
Apln
Tlr2
Esyt2
Aida
Atp11b
Rin2
Wnt16
Zfp518a
Cenpf
Nt5e
Meox2
Ptgfrn
Usp6nl
Pxdn
Akt3
Zfp677
Galnt4
Abhd2
Osbpl11
Frk
Stk3
Slc30a5
Pabpc4l
Fig4
Kdm4c
Scn7a
Stam
Nbas
Timm50
Irf2bp1
Lrg1
Ypel3
Fkrp
Fen1
Wdtc1
Ppp3cb
Rps6kb2
Mrpl4
Sac3d1
Scmh1
Ino80e
E2f3
Spsb3
Agpat1
Parp16
Rnpepl1
Hmg20b
Mical2
Hspbp1
Stab2
Rnf19b
Ighmbp2
Slc25a44
Phpt1
Rtn2
Vrk3
Top1mt
Speg
Pofut2
Rxrb
Tom1l2
Por
Gps2
Mark2
Wbp11
Dlgap4
Hagh
Psen2
Nfic
Msl1
Nub1
Grina
Gga1
Pcid2
Apex1
Shisa4
Cox10
Med25
Dnajc30
Txn2
Dbp
AI837181
E2f4
Mon1a
Pik3r2

Spata7	Abcb1b	Ctdsp1	Comp	Znhit1
Tipin	Syk	Slc19a1	Thsd4	Exosc5
Ube2j2	Nvl	Ciz1	Arl15	Tomm40l
Ankra2	Apold1	Anapc13	Fbxo30	Impdh1
Slc15a4	Abra	Poll	Slc25a46	Gck
Urod	Snrnp200	Rnpepl1	Atad1	Usp10
Cpe	Rhpn2	Sema6c	Nup153	Zfpl1
Mrps14	Il1rl1	Mgat4b	Plce1	Prkag2
Rit1	Prps2	Ets2	Dock8	Fis1
Fam162a	Timp1	Calr	Glce	Mpg
Klhl21	Cilp2	Snrpn	Epha3	Lrrc14b
Plcl2	Zbtb6	Gnb2	Arl5a	Fam50a
Cetn2	Tgfbr2	Cuedc1	Fam3c	Pcbp4
Homer1	Gulp1	Sgsm3	Adamts12	Tada3
Tas1r1	Kdm3a	Hhatl	Bdh2	Vgll4
Mogs	Hspa5	Slc29a2	Nup205	Rbm38
E2f5	Il10ra	Ybx2	Zfp397	Map3k14
Atf4	Haus2	Gpr108	Actr3	Eif3f
Nenf	Dmxl2	Scmh1	Ccl9	Amhr2
Ddrgk1	Nt5e	Rtn2	Zfp955a	Smap1
Gmip	Kif16b	Wdr77	Tlr8	Rai2
Rp9	F5	Hoxa7	Abca1	Ip6k2
Pla1a	Rap1gap2	Gmppa	Anxa5	Armc5
Hmgcl	Krit1	Ptrh1	Il2	Slc35c1
Psip1	Eri1	Ddx39b	Adam10	Lrtm2
Camk1	Slc15a2	Srrd	Pibf1	Eef2k
Rexo4	Zfp518b	Eif3g	Chodl	Mef2d
Eif4a3	Rfwd3	Cacng1	Ube3c	Zkscan17
Gtf2h2	Pkd2	Cs	9130019O22Rik	Cd74
Zfp639	Slc35f1	Dnmbp	Lepr	Setd7
Orai1	Ddr2	Mapkapk3	Gnai3	Prpsap1
Dynll1	Atr	Mrpl52	Zfp27	Hgs
Hmg20a	Zfp37	Tmub2	Cysltr1	Mfn2
Ccdc43	Hace1	Timm8b	Atf3	Hps6
Ctla2a	Ctnna1	Elmo2	Anxa3	Csnk1e
Frg1	Naip5	Pitpna	Snx14	Wnk2
Ccdc88c	Vps4b	Cdipt	Capn7	Ttf2
Chchd2	Tbl2	Rab40c	Snx7	Ankrd54
Dpep1	Ano3	Pigt	Cfh	Mrpl52
Pstk	Exoc8	Gck	Msr1	Mrpl55
Fez2	Pitx2	Usf2	Urb1	Rexo2
Ppp2r2d	Lyst	Rbm22	Ttc38	Phc1
Arfip1	C1qtnf3	Tmem109	Rab33b	Pcyt1a
Cox6a1	Il13ra1	Fxyd1	Pole	Parp6
Fam161b	Lgr5	Exosc5	Zfp933	Actr1b
Smox	Scamp1	Rgma	Cdkn2aip	Alkbh7
Cdc26	Ppm1k	Eif4ebp1	Topbp1	Calr
Yrdc	Klhl32	Dhcr7	Plxdc2	Ctsb
Fastkd3	Zfp658	Abcd1	Trpv2	Trim7
Unc119b	Myo1e	Smtnl2	Gnb4	Zfp689
Mzt2	Pls3	Pih1d1	Ezr	Gorasp2
Taf5l	Gpbp1	Scand1	Tgfbr2	2300009A05Rik
Eif2d	Zfp60	Dhdh	Cgnl1	Terf2
Tmem14c	Zfp442	Slc7a8	Papss2	Ctnnbip1
Mrps16	Ttc39b	Tmem52	Birc3	Satb1
Aurka	Gpr34	Sf3a2	Postn	Zyx
Fcf1	Tnfaip6	Smap1	Appbp2	Plekhj1

Commd1
Cox4i1
Hyal2
Siva1
Pex7
Fn3k
Med20
Rpl22
Rchy1
Snrnp35
Atp5e
Tcea2
Ndufa13
Rnf157
Mpzl3
Egflam
Ndufb5
Ssna1
Rpl26
Zfp786
Scfd2
Ndufb7
Hspb11
Kptn
Rpp40
Swi5
Cd14
Prkab2
Psmc3
Ptpmt1
Lrtm2
Ppp1ca
Mrps30
Smarcb1
Fahd2a
Shisa2
Dusp28
Pdhx
Surf6
Rnf34
Tek
Zfp507
Gas2
Usp8
Olfml2b
Stk38l
Tlr8
Tbc1d12
Rlim
AI597479
Slfn5
Ptgfr
Cd163
Btaf1
Dido1
Lamc2
Kif20b
Jag1
Loxl3
Iqgap2
Clasp2
Ddx42
Rgs4
Dock2
Klhl23
Fmr1
Vps13c
Fn1
Ncf2
Wwtr1
Zfp182
Chrna1
Comp
Mgat2
Casd1
Trim44
Plek
Mfap1b
Sec24d
Ankle2
Lamc1
Slc30a4
Sgpl1
Fpgt
Ggta1
Arhgap24
Kdm4c
Nucb2
Cpd
Fsd1l
B3galt2
Hsp90aa1
Sar1a
Cep170
Pcdh20
Ttc26
Utrn
Mrpl34
Hcfc1r1
Mrpl36
Mapk1ip1
Sox18
Atp13a1
Tnip1
Slc25a42
Rabggta
Cops7a
Atat1
Ptms
Pkig
Chchd10
Ssbp4
Alkbh6
Rab7
Pcbp2
Gja4
Spata2l
Ap4b1
Tom1l2
Ndrg3
Tusc2
Sap30l
Hlx
Satb1
Mark2
Zfp691
Ctbp2
4930432K21Rik
Cnot3
Rab35
Tgfb1i1
Ly6e
Dbp
Kdelr1
Zmiz2
Eif4ebp2
Tob1
Ctbp1
Trp53
Phb2
Tfap4
Rac1
Blcap
Lage3
Acp6
Polrmt
Irak1
Dusp22
Angel1
Synpo2l
Lyrm7
Mier2
Rnf14
Sesn2
Rnf4
Apaf1
Pcdhb16
Bcat1
Acvrl1
Cdk15
Mis18bp1
Srpx2
Top2a
2610008E11Rik
Zfp606
Steap2
Tjp2
Txlng
Zfp759
Mrc2
Ckb
Kcnk2
Epha4
Lims1
Ints8
Cyth4
Strn
Tnpo1
Pigk
Fam167a
Rbbp8
Plekhh2
Ptprc
Pkd2
Ccnc
Abcb1a
Rpl7l1
Akap8
Ccdc80
Tnmd
Lin7a
Cdk17
Zfp449
Clec1a
Col11a2
Gmfb
Vrk1
Zfp202
Lrrk2
Tlr4
Dhx57
Exoc1
Dgkb
Zfp11
Boc
Abi3bp
Pcdh20
Tspyl5
Zfp68
Galnt11
Zfp52
Syvn1
Kctd13
Snrpc
Ssu72
Mafb
Acsf3
Fam3a
Rnf5
Gtf3c2
Ccdc6
Map2k2
Zmynd11
Gpx3
Ube2f
Trp53inp2
Tmcc2
Dazap2
Map2k5
Mbd1
Nrarp
Fam53b
Rilp
Cox17
Dtnbp1
Fbxl8
Wrap53
Atp5d
Zfp553
Ube2d1
Adcy9
Samd10
Bcdin3d
R3hdm2
Fam122b
Ascc1
Usp2
Nfatc1
Mrpl24
Zfp444
Hdac5
Eif2b2
Cc2d1b
Tpm2
Serhl
Cyc1
Tspo
Rbm24
Rgma
Zbtb45
Ap1s1
Sspn
Trim32
Dab2ip
Tor1aip1
Stk40
Ctbp1
Grwd1

Gas2l3	Rplp1	Uba5	Snrpb
Vav3	Nudt16l1	Il4ra	Zfp408
Abcd3	Rnf214	Hecw2	4931414P19Rik
Necab1	Ccdc86	Klhl5	Banf1
Esco1	Nfatc1	Grpel2	Urm1
Cmah	Slc35a4	Yipf6	Chchd7
Slc30a7	Mfsd5	Slc15a2	Gtpbp6
Bmp3	Rab2b	Epsti1	Rcsd1
Gmps	Prickle3	Abca5	Fto
Lamb1	Dctn2	Dock9	Pex11b
Mfsd6	Eif1b	Stt3b	Nr1h3
Ctsl	Zfp362	Snip1	Slc5a6
Fndc3a	Dlgap4	B3galt2	Fam160b2
Col4a5	Sh2b3	Prkch	Nt5m
Ptprd	Gpc1	Cyp2j6	Lmo4
Pdia4	Trim11	Pcmtd2	Slc45a4
Herc4	AU040320	Pla2g4a	Pou6f1
Fam126a	Aip	Dact2	Tysnd1
Rab3gap2	Ache	Adam22	Mknk1
Nbas	Tbrg4	Fbxo40	Sdr39u1
Thsd4	Nfkbib	Tgoln1	Zfp36l1
Hecw2	Zer1	Nudcd1	Podn
Pxdn	Setd7	Myh11	Cs
F13a1	Tbc1d25	Olfml2a	Vasp
Angpt2	Rhbdd3	Canx	B3gat3
Hltf	Tnfrsf23	Tmem181a	Pdgfb
Agps	Nckipsd	Adh7	Cdk20
Cdc42ep4	Map2k4	Atp8b2	Sema6c
Ccl9	Mdh2	Ephb2	Prickle3
Smarca5	Nfix	Zfp946	Snrpd2
Zfp157	Preb	Btg2	Sult5a1
Mdga2	Ubap2	Stk17b	Mybbp1a
Fam107b	Hsf4	Itga2	Rnf220
Zc3h14	C2cd2l	Mfap1b	Spns1
Copb1	Fkbp1a	Prex2	Ndufs7
Fam102b	Zfp414	Lrch1	Pgp
P4hb	Cops8	Atp6ap2	Ank1
Lars	Usp21	Cab39	Gsk3a
Gcc1	Car14	Samsn1	Tbc1d9b
Gria3	Dcakd	Klhl7	Dnajb5
Col11a2	Hoxa4	Zfp790	Rnpep
Tec	Acot8	Heatr5b	Csnk2b
Wdr19	Chac1	Fstl1	Tmem201
Zfp760	Ramp2	Sesn1	Cpsf7
Rin2	Apex1	Lrba	Gnb2
Pde3a	Dpp9	Fbln2	Helt
Rassf4	Klc2	Thbs1	Plscr3
Itga11	Snrpa	Anxa4	Mlf2
Vps26a	Ppme1	Gmps	Ywhag
Trim37	Sf1	Ankrd13a	Dpm1
Pgm3	Nrip2	Cstf2	Tial1
Hepacam2	Ufsp1	Was	Ltbr
Phldb2	Vkorc1	Loxl2	Nfix
Cul4b	Dolpp1	Pnpt1	Fitm1
Arhgap18	Rnf157	Sphk1	Six5
Fktn	Tfeb	Dstn	Prox2
Plekhh2	Ulk1	Prkdc	Htra3

Sdhaf2	Adpgk	Vma21	Eef1a2
Prkg1	Por	Man2a1	Ilk
Yipf6	Ccdc97	Tmem18	Hhatl
Zfp850	Klhl30	Zfp111	Me1
Caprin1	Dok7	Ddx58	Inppl1
Pbld2	Trak1	Ivns1abp	Ppp1r18
Fam135a	Ccnf	Gpm6b	Trip10
Gpsm2	Ampd2	Zfp874a	Scamp2
Inpp1	AI413582	Pstpip1	Plekhb1
Zfp773	Sh2d3c	Syap1	Fam193b
Esyt2	Ormdl2	Slc9a9	Parp3
Clcn5	Wbp11	Rnf213	Ube2l3
Zfp944	Golph3	Zfp810	Fam131a
Adamts20	Cox11	Zfp939	Map2k4
Ctnnb1	Rps5	Mcm6	Eif4ebp2
Stk17b	Htra3	Mcm4	Eif6
Hacl1	Ell	Magi1	Wars
Klhl13	Bcl2l12	Itgav	Rnf44
Yeats2	Asnsd1	Frmd6	Ung
Lrrcc1	Lsm2	Cep76	Sh3bp2
Fam76b	Rxrg	P2ry1	Akr7a5
Arhgap11a	Rapgef1	F3	Aup1
Myh10	Rbpms	Msh3	Tpra1
Mthfd1	Dctn6	Trdmt1	Ubqln2
Klhl9	Zyx	Rap1b	Thap1
Kcnk2	Adck2	Cd38	Camk2b
Col12a1	Gtpbp6	Gas2	Hs6st1
Nek1	Ces1d	Arf6	Wdr77
Cilp	Otub1	Vwa5a	Kcnc4
Shroom4	Phf1	Wdr19	Adprh
Sepsecs	Ccdc84	Sdcbp	Endog
Ift172	Stk40	Scx	Nrip2
Rbm41	Rab11b	Pi15	Ogfod2
2210408I21Rik	Slc25a37	Fam76b	Hsf1
Wrn	Clybl	Tec	Ntf5
Klhdc1	Suv39h1	Prex1	Des
Eprs	Vrk3	Cd34	Sap30l
Cyth4	Btbd2	Nphp3	Bhlhe41
Slc37a2	Tjap1	Fes	Tmem107
Matn4	Btbd6	Lrp12	Cpox
Cerk	Camta2	Pi4k2b	H2-Ab1
Dcp1a	Tbc1d10b	Copb1	Shkbp1
Mbtps1	Bak1	Tigd4	Pcgf2
P2ry12	Srsf2	Dip2a	Sim2
Cab39	Lamp1	Tbc1d8b	Opa3
Brip1	Rasa4	Cacna1g	Ppif
Zc3hav1	Gnai2	Igfbp6	Tbc1d25
Sdc4	Chmp4b	Acvr2a	Klhdc3
Usp15	Mrps34	P2ry12	Ciz1
Ildr2	Srebf1	Emb	Ube2r2
Tiam2	Sfxn3	Yes1	Ccnk
Zfp202	Plaur	Cd53	Dedd
Zfp759	Trim8	Igsf6	Hoxc4
Tfrc	Ccm2	Cdc27	Arl2
Ttl	Peli3	Maff	Ap4b1
Cdkn2aip	Crip3	Mapk8	Carm1
Stard4	Dusp7	Casp8	Mrps18c

Fbln2	Acsf3	Tpp2	Myadml2
Rnf2	Atf1	Nlrp10	Thrsp
2610008E11Rik	Prpsap1	Col22a1	Camk2g
Nedd1	H2-Ab1	Egfr	Prdx5
Otud6b	Cox8b	Zbtb6	Park7
Fxr1	Tbc1d17	Foxn2	Rasl12
Spock3	Rhot2	Xcr1	Cdk2ap2
Msh6	Hoga1	Ercc6	S100a4
Aspm	Josd2	Sptlc2	Mypn
Pex13	Thap1	Tdp2	Zmiz2
Dcp2	Tmem63a	P4ha1	Znrf1
Emp1	Arid3b	Naip5	Kcnq4
Lzts1	Gpank1	Atad2	Taf1c
Frmd6	Trp53inp2	Abat	Ulk1
Osbpl11	Slc35c1	Gbp3	Cops7b
Eif2ak2	Pml	Smo	Khsrp
Bzw1	Mpz	Kdm3a	Dennd1a
Gprasp1	Rnf44	Smarce1	Sirt7
Dync2h1	Hlf	Ifit3	Plvap
Lca5	Smn1	Rbm41	Add1
Brox	Slc35b4	Zkscan1	Ctdsp1
Ythdf3	Pgs1	Itch	Bpgm
Rad51c	Nub1	Zfp157	Rap1gds1
Ipo7	Stk35	Myh7	H6pd
Arhgef40	Map3k3	Mob1a	Cdipt
Rps6kc1	Bmyc	Kif11	Tmem109
Far1	Slc45a4	Ddr2	Atp1b2
Ascc3	Pelp1	Tlr7	Eif4e2
Cdh2	Mrpl10	Slc30a4	Spata2l
Eif1a	Hba-a2	Gosr1	Nlrx1
Ttc21b	Hs6st1	Ythdf3	Ptms
Atrn	Lrrc56	Fam114a1	Thap7
Cmpk2	Acvr2b	Mpv17l	Dda1
Atp9b	Mettl1	Ntn4	Fbxo6
Slc35a3	Ube2r2	Uba2	Acin1
Cdk15	Arhgdia	Acsl5	Dph2
Rasa3	Lrrc61	Pds5a	Scamp3
Snx14	Ehd1	Zfp266	Pex12
Myh11	Ttll12	Pold3	Timm17b
Tll1	Anapc11	Enox1	Bop1
Fam120b	Cand2	Zfp882	Flii
Baz1a	Comt	Stk38l	Fkbp1a
Zfp882	Ckm	Zfp128	Dnmbp
Tanc1	Hoxb4	Gm14305	Atg4d
Ppfibp1	Gpn2	Loxl3	Ptp4a3
Kif23	Pxn	Klhl13	Fbxw4
Vps35	Zfp444	Nr5a2	Dmwd
Matr3	Dcaf15	Suclg2	Rp9
Ctsk	Atp5j	Armcx3	Agap3
Ccdc14	Zdhhc4	Ythdc2	Dgcr6
Thbs2	Parp3	4933427D14Rik	Mrpl36
Tlr13	Arpc4	Sdc2	Ybx2
Zfp568	Ksr1	Copa	Gstcd
Tjp1	Hipk2	Plek	Fbxl16
Ankrd50	Rorc	Slc5a3	Tpp1
Utp18	Tprgl	Gpkow	Tmem160
Zdhhc20	Tpcn1	2210408I21Rik	Slc25a37

Emilin1
Zfp951
Asb1
Mrc2
Prkd3
Zfp947
Topors
Pigk
Dixdc1
Eml4
Trim23
Duox1
Tnpo1
Cast
Smc6
Nid1
Ppp6r3
Otud1
Hmmr
Xcr1
Pja2
Haus8
Ipo5
Armcx3
Atxn3
Clec14a
Ccnc
Mpp7
Usp40
Parp9
Cep76
Zscan18
Rbm4b
Itgam
Sdcbp
Add3
Cd44
Steap2
Npr3
F2r
Myh3
Actr3
Dgkb
Nlrc3
Bche
Smc3
Tmtc3
Capn3
Jrk
Gpr153
Pank3
Prtg
Mre11a
Htt
Mis18bp1
Nbea
Pygb
Sec24c
Ovca2
Tmem147
Thtpa
Mrpl14
Fen1
Prkaca
Rela
Mapre2
Strada
Pld2
Ppp1r14c
Vgll2
Lin52
Ssr4
Rfx1
Zfp579
Emd
Dazap1
Slc35c2
Jagn1
Hnrnpul1
Tbc1d9b
Ndufb11
Nprl3
Timm17b
Sox13
Cited2
Pfkfb1
Tst
Tmbim4
Zswim1
Ubl7
Ccdc85c
Stip1
Il17rc
Cln6
Rnf8
Cnksr1
Maz
Mmp15
Speg
Slc25a27
Cdk16
Znrf1
Acd
Carns1
Msrb2
Lgals3bp
Rbm24
Slc9a1
Syngr2
Ptp4a3
Bcar1
Gga3
Coro2b
B4galt1
Klhl4
Rras2
Kif23
Ptpn7
Mapk10
Fgl2
Chrna1
Ankle2
Serpinb8
Cog3
Arf2
Fbln7
Ptpn12
Syk
C330007P06Rik
Plaa
F5
Aldh3b1
Iqgap2
Dtx4
Plekha5
A430033K04Rik
Fgf7
Actr2
Tceal8
Ptafr
Cmpk1
Lrpap1
Cnot1
Sh3d19
C1qtnf2
Zfp442
Zfp953
Osbpl8
Dars
Slc44a1
Copb2
Nudt12
Lrig2
Galnt5
Cd93
P2rx4
Sepsecs
Trp53inp1
Gopc
Phldb2
Endod1
St8sia1
Tmed5
Casd1
Tmod2
Reln
Tmx4
Il1rap
Irf4
Aspm
Ikbip
Ext2
Slc7a6os
Ints5
Elof1
Ccdc163
Mllt11
Anapc13
Gpbp1l1
Rap1gap
Mapre3
Polr3c
Rbm20
Eif3g
Brd3
Prrg2
Crip3
Entpd6
Fam78a
Cuta
Tmem140
Chmp4b
Ssr4
Plekhm2
Habp4
Rprd1b
Dpf2
Pgrmc2
Tab2
Iqsec1
Tmub2
Pcbp2
Plekhb2
Amz2
Tspan7
Tmem216

Uba3	Fhod1	Cycs
Ralgps2	Tbc1d22b	Pik3c2a
Lrp4	Cntfr	Xab2
Lhfp	Gga1	Slc6a4
Kdm4a	Trpt1	Larp6
Arhgef10	Ube2e2	Cth
Zfp329	Cuedc2	Cmah
Casp1	Fbxl8	Dsel
Abca6	Atxn2	Ttc26
Gja1	Tnnc2	Adam9
Prickle1	Stub1	Tmem182
Atad1	Pabpc1	Robo1
Itgav	2300009A05Rik	Ascc3
Ccdc80	Herpud1	Cnn2
Txlna	Traf3ip2	Unc13c
Rabgap1	Hs1bp3	Myo9b
Smc5	Sufu	Btaf1
Arhgap6	Pdgfa	Zfp719
Sf3b1	Wdr62	Fzd6
Fat3	Nr1d1	Sod2
Oaf	Rasd2	Hspa5
Armcx4	Setd6	Col23a1
Bmpr1b	Thyn1	Sephs1
Kdr	Gprc5b	Alms1
Loxl4	Dgcr6	Tes
Clpx	Odf3l2	Tmem117
Tmx3	Fbxw5	Amotl2
Pfkp	Tmem59	Arcn1
Lox	Spsb3	Scyl2
Zfp119a	Rara	Abca9
Mynn	Camk2b	Tgfbr1
Smchd1	Srsf9	Zfp748
Trip4	Tmem176a	Rnf149
Cdh11	Shkbp1	Ubash3b
Anxa3	Ercc2	Ctsk
Nars	Pdgfb	Mthfd2l
Tax1bp1	Mfn2	B4galt4
Sparcl1	Slc44a2	Adk
Picalm	Habp4	Plod1
Scai	Tead3	Nsa2
Dnmt1	Gtf3a	Mill2
Ints8	Mocs3	Slc9a6
Taf1	Ints3	Nt5c2
Eif3c	Rhbdl3	Plcxd2
Cybrd1	Fbxl16	6720489N17Rik
Atp8b2	Srsf4	Caprin1
Abhd13	Fbxw9	Zfp760
Lamb2	Arfgap2	Trappc8
Dars	Add1	Olfml3
Zfx	Atxn2l	Ppl
Ankrd1	Slc6a9	Nars2
Entpd5	Tmem11	Tnfaip6
Ybey	Tnk2	Dmxl2
Calcrl	Sugp1	Arhgap6
Fam167a	Timm50	Cops2
Ano1	Sac3d1	Bcl6b
Ccar1	Med15	Rrm1

Nbn	Med19	Zfp563
Dclk2	Tmbim1	Pggt1b
Tbc1d2b	Tmem9	Rnd1
Clec5a	Crtc3	Zfp951
Polr2b	H6pd	Tbc1d23
Galnt11	Zfp598	Npat
Wif1	Pnrc1	Oit3
Junb	Mafg	Sgk3
Rabgap1l	Trim28	Hccs
Pik3c2a	Ergic1	Klhl6
Wdr76	Med26	Il6st
Abca8a	Mapk11	Ankrd28
Scx	Tmem184b	Atg2b
Spon2	Dbi	Mdga2
Il17rd	Bbc3	Cd300a
Twsg1	Ube4b	Kcnt2
Arhgap12	Sgta	Lpar1
Iqgap1	Nol6	Cd300lb
AW549877	Smad3	Acp1
Pja1	Snrpf	Hnrnpa3
Asap3	Ext2	Shroom4
Zfp105	Tysnd1	Gns
Stk4	Tmem201	Ptprr
Usp6nl	Paqr3	Foxred2
Srfbp1	Tubg1	Ncapg2
Bace2	Sctr	Efemp1
Ercc6	Phf2	Zfp101
Trpv2	Dcaf6	Hsp90aa1
Enox1	Hagh	Mmgt1
Scara3	Ppard	Srfbp1
Qpct	Dmwd	Sdc1
Ercc6l	Limk1	Ifnar1
Gufl	Riiad1	Rad51c
Ctnnal1	Dynlrb1	Heg1
Fcrls	Cdk10	Pigg
Pold3	Ssr2	Bace2
Btg2	Zdhhc1	Exoc8
Wdr3	Tial1	Nck1
Gda	Cc2d1a	Il16
Lcorl	Timp3	Lyve1
Ppp1r16b	Pcbd2	Parva
Larp7	Mllt1	Cap2
Ubxn7	Vezf1	Zfp37
Tmod2	Ptpmt1	
Sv2c	Ctf1	
Gfpt2	Tmem219	
Cnst	Plekhh3	
Ptpra	Sorbs3	
Evi5	Nr1h3	
Prkch	Zfp689	
Nedd4	Bag3	
Map3k2	Cope	
Itpripl2	Ap1g2	
Cysltr1	Phkg2	
Cc2d2a	Map2k5	
Urb1	Cds1	
Zkscan5	Fbxw4	

Plekha5	Pcbp3
Zfp799	Fance
Rgs17	Rhbdf2
Ptk7	Baiap2
Zfp874a	Park7
Dhx29	Arpc2
Hexa	Dpm1
Zfp958	Zcrb1
P2rx4	Sec14l1
Tubb5	Slc25a35
Calu	Prrc2a
Cntln	Grn
Spin4	Slc36a1
Zfp40	Des
D6Wsu163e	Trmt1
Nhlrc3	Dpm2
Etaa1	Vamp2
Lysmd3	Lmo4
Amotl2	Prr14
Dclre1a	Mea1
Mtx3	Gdpd5
Ankib1	Pcgf1
Abcb7	Neurl2
Tanc2	Capzb
Ryr3	Ppp1r16a
Rnf20	Brpf3
Pecam1	Slc38a7
Irak4	Fbxl6
Prpf6	Cux1
Cytip	Stap2
Tceal8	Elk1
Tmem119	Mrto4
Cpm	Rnf10
Eif5a2	Fam193b
Nampt	Thra
Snx25	Napa
Map4k3	Adra1a
Actr2	Actr1b
C4b	Cmtm7
N4bp2	Snx15
Plin4	Rogdi
Itgb2	Ecm1
Ccdc8	Gnptg
Dennd4a	Cacng7
Nup155	Map2k6
Zfp52	Sh3bp1
Ivns1abp	Psmd11
Erbb4	Cited4
Trim2	Mark4
Fbxo40	Psap
Cstf2	Mmab
Ric3	Irf2
Hif1a	Armc6
Vopp1	Ranbp3
Tjp2	Tdp1
Ptafr	Abcc10
Zfp273	Dtx3

Lrrn4cl	Nfu1
1700019D03Rik	Arl8a
Myo7a	Gemin8
Lpar4	Pim2
Nsun7	Lrrc8a
Ahnak2	Eif2b2
Fnip2	Tmem141
Cdon	Gnb3
Tbx18	Thrsp
Chid1	Mbd6
Adh7	Mnt
Creb1	Ebf3
Ints2	Mad2l2
Col6a3	Atg13
Dnm1l	Mrpl45
Rras2	Nfkbil1
Plaa	Fscn1
Mertk	Fbrs
Kif3b	Zfp341
Zfp62	Fzd7
Epdr1	Hdac7
Tceanc	Rab4a
Atm	Bola3
Helb	Polm
Pola1	Whamm
Cadps2	Gpbp1l1
Mthfd2	Phf21a
Hspa4	Gse1
Pdlim5	Nt5dc3
Mios	Pmf1
Snx33	Sh2b1
Uap1	Cpne2
Dtx3l	Cdc42ep1
Rnf13	Pecr
Tubgcp5	Fzr1
Ntrk2	Tap1
Larp4	Psmd3
Mvp	Lrtm2
Pcdhb17	Ino80b
Rab8b	Cpsf7
Ifnar1	Copz2
P4ha1	Klf13
Mttp	Pou6f1
Abi2	Mapk3
Ttc38	Tada2b
Arf6	Parvb
Myh6	Letmd1
Ptpn12	Dcaf12
Smc2	Git1
P2ry10	Sp2
Dgke	C1qb
Itch	Tbc1d7
Ppil4	Ubap2l
Pcdhb22	Bola1
Mthfd2l	Arc
Snx5	Alg3
Stap1	Cyc1

Wdr37	Prcc
Cpsf2	Med9
Arf2	Rhog
Zfp120	Ncln
Pus10	Tacc3
Cdkl3	Fbxl12
Amot	Map3k10
Crnkl1	Daxx
Tmed8	Ccdc134
Ccr5	Zfp710
Sh3bgrl	Tomm40
Osbpl2	Idua
Metrnl	Acot7
Flrt3	Ociad2
Flrt2	Dab2ip
Cep135	Neurl1a
Nusap1	Det1
Chmp3	Mlst8
Wdr78	Dgkz
Socs6	Atf6b
Slc25a20	Tcf3
Fancm	Fiz1
Mis12	Pim3
Tlr3	Repin1
Nsl1	Dusp13
Tdrd7	Pebp1
Fam199x	Fdps
Rock1	Asb6
Zfp68	Srf
Ddx31	Dusp8
Gxylt1	Pcgf2
Gzf1	Icosl
Mafk	
Pdk3	
Sdc2	
Zfp738	
Adamtsl2	
Scn7a	
Rapgef4	
Rnf213	
Tbxas1	
Rad50	
Fbxo3	
Ctdspl2	
Ica1	
Greb1l	
Ptdss1	
Golga1	
Hmcn1	
Mospd2	
Nbr1	
Hmgcs1	
Zfp455	
Gprasp2	
Acvr2a	
Gm12185	
Nrp2	

Prkg2
Col5a1
Ntm
Iws1
Cul1
Hs2st1
Sf3a3
Cxxc4
Ddx20
Dnajb4
Zfp719
Ccdc62
Zmynd8
Maged1
Snx13
Zfp54
Exoc2
Cpt1a
Adamts8
Dhx8
Xrn2
Zfp111
Nup133
Zmym6
Cdk7
Pdik1l
Gtf3c4
Reln
Nr3c1
Mtm1
Dcaf10
Lpar1
Xpc
Exoc5
Hspa4l
Pwp2
Casp8ap2
Zfp763
Tusc3
Capn7
Zfp292
Scin
Scyl2
Trim33
Inpp4b
Gpd1l
6720489N17Rik
Canx
Ahi1
Fbxw7
Vrk1
Zfp407
Gbp9
Zfp266
Wwp2
Suv39h2
Etf1

Atp11b
Gabpb2
Ace2
Ptgis
Klhl15
Atf3
